# Supplementary material for: Discovery of a Novel DNA Gyrase-Targeting Antibiotic through the Chemical Perturbation of Streptomyces venezuelae Sporulation
Source: Cell Chem Biol. 2019 Sep 19;26(9):1274–1282.e4. doi: 10.1016/j.chembiol.2019.06.002 (PMC6856721; doi:10.1016/j.chembiol.2019.06.002)
Supplement: Document S1. Figures S1–S5 and Table S1 [file mmc1.pdf]

Cell Chemical Biology, Volume *26*

Supplemental Information

Discovery of a Novel DNA Gyrase-Targeting Antibiotic through the Chemical Perturbation of *Streptomyces venezuelae* Sporulation

Scott McAuley, Alan Huynh, Alison Howells, Chris Walpole, Anthony Maxwell, and Justin R. Nodwell

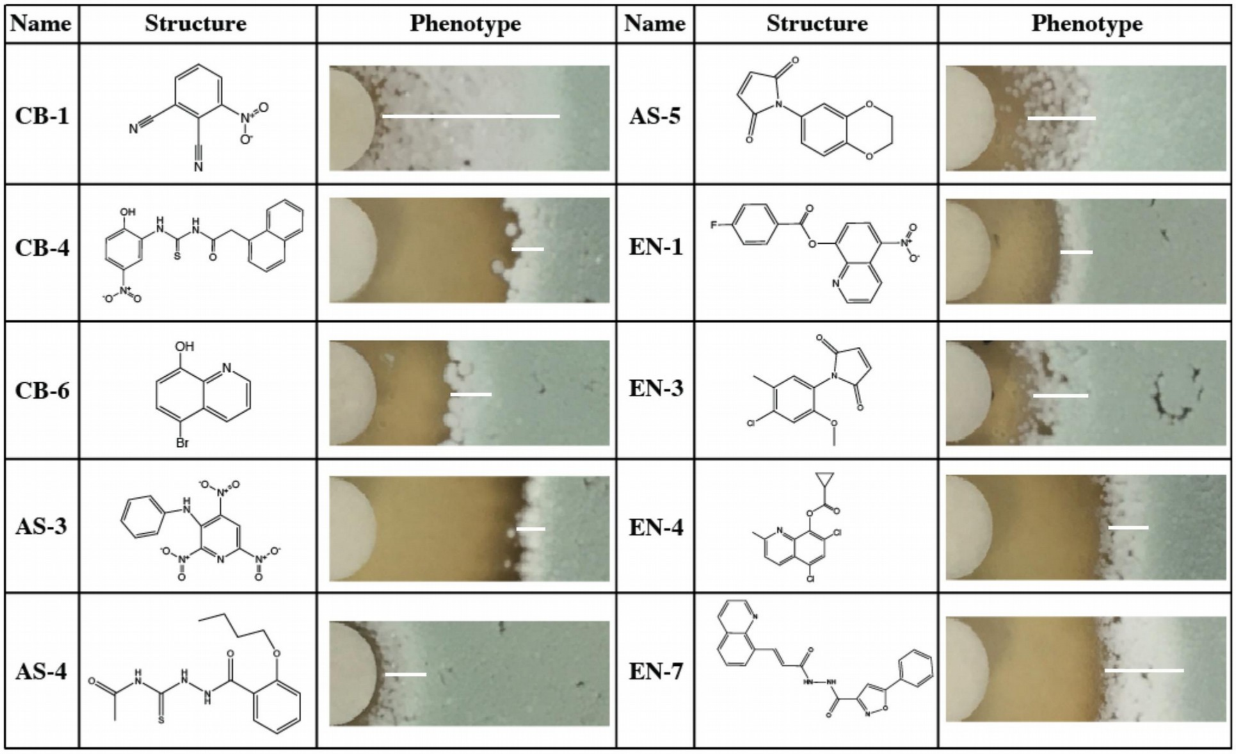

Figure S1, related to Figure 1. Chemical structures and inhibition of *S. venezuelae* sporulation by the screening hits. 10 µL of each molecule was added to the drug disk, placed on a lawn of *S. venezuelae* spores and incubated at 30 ^{o}C for 48 hours before imaging. The EN-1 and AS-5 stocks were 10 mM while the remaining were at 2.5 mM. A white horizontal line has been added for visual reference.

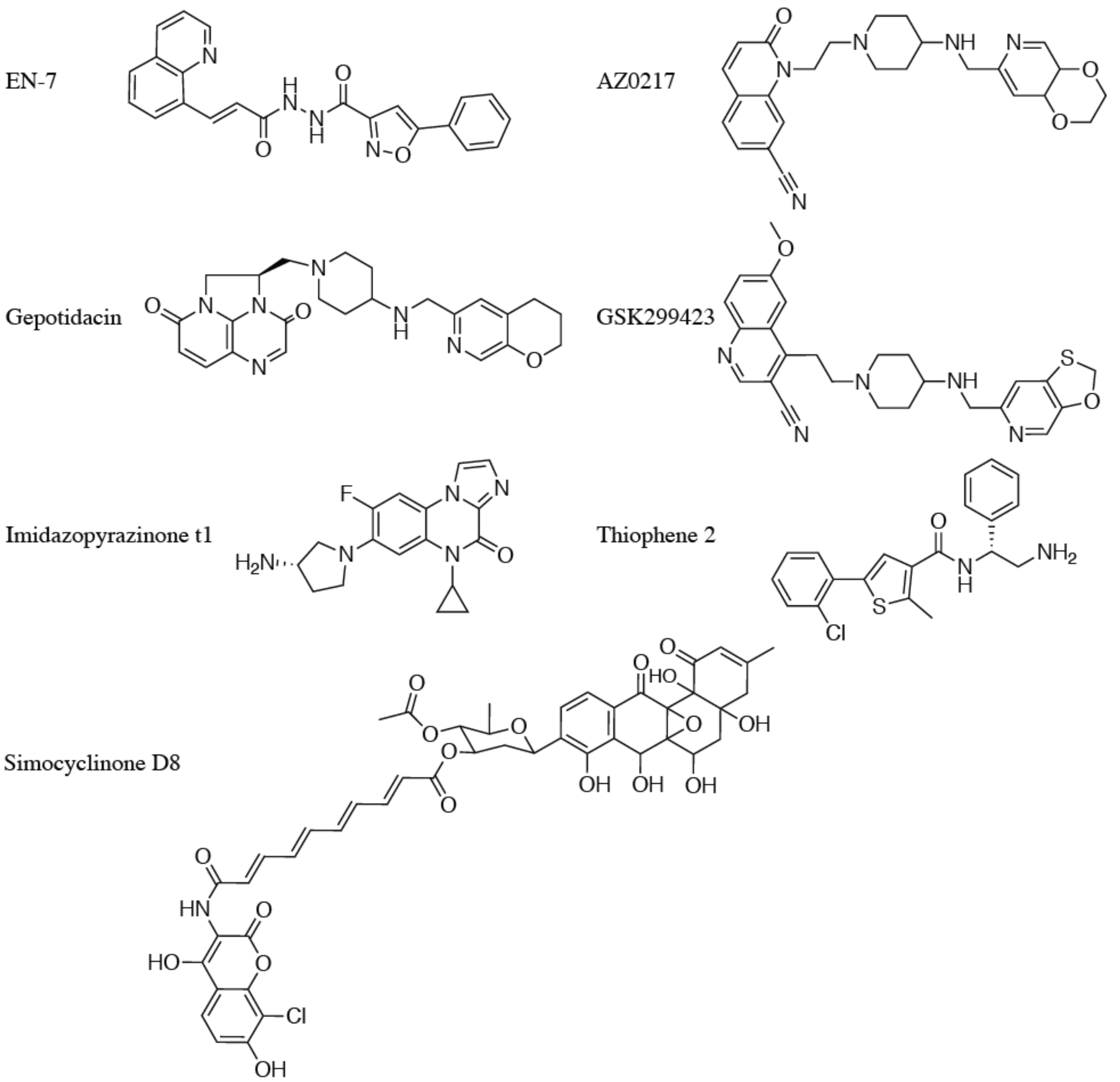

Figure S2, related to Figure 2. Chemical structures of EN-7 with other investigational gyrase inhibitors

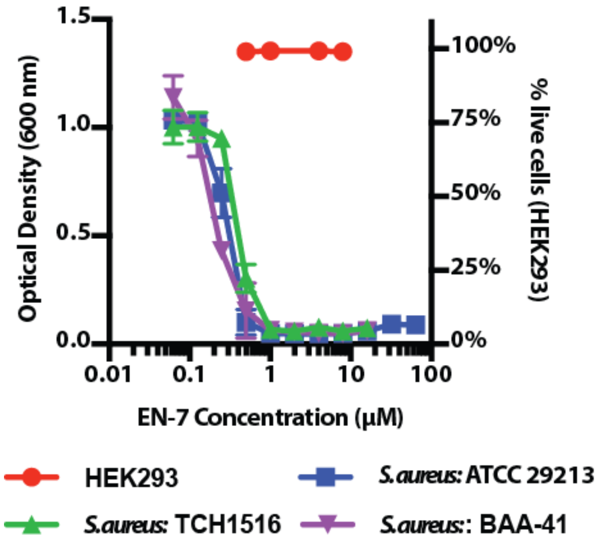

Figure S3, related to Table 1. EN-7 inhibits growth of Gram-positive *S. aureus* while having no effect on human cell lines, such as HEK293.

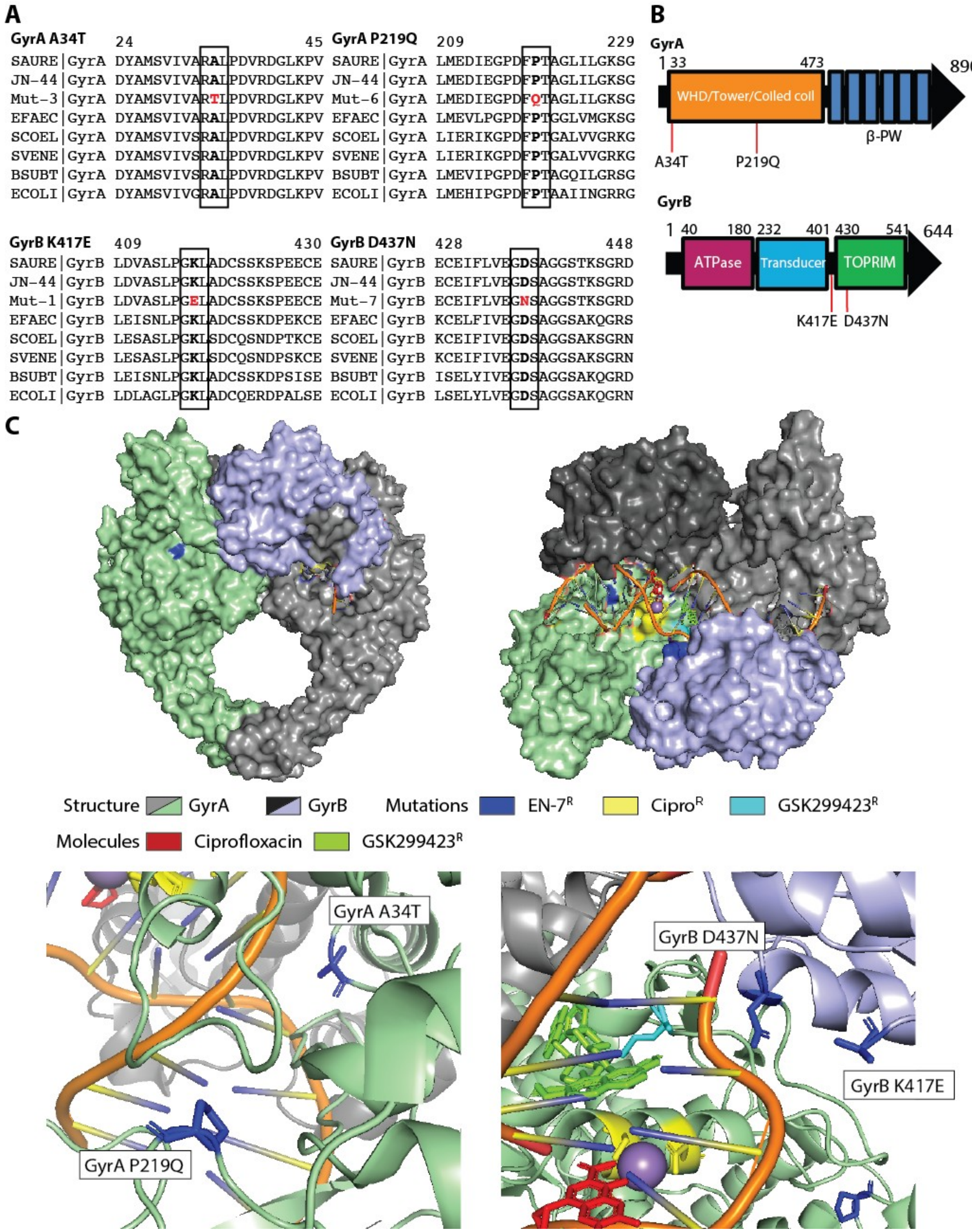

Figure S4, related to Table 2. Analysis of EN-7-resistance gyrase mutations. (A) Amino acid alignment of the two GyrA and two GyrB mutations found in the EN-7 resistant *S. aureus* strains against published *S. aureus* (SAURE), parent strain (JN-44), *Enterococcus faecalis* (EFAEC), *Streptomyces coelicolor* (SCOEL),

Streptomyces venezuale (SVENE), *Bacillus subtilis* (BSUBT), and *Escherichia coli* (ECOLI) sequences. (B) Location of EN-7 resistant mutations on linear GyrA and GyrB domain structure. (C) Location of EN-7 resistant mutations mapped to a published gyrase crystal structure. The structural information was created by overlaying a *S. aureus* gyrase structure with GSK299423 (2XCS) and a *S. aureus* gyrase structure with ciprofloxacin (2XCT). Ciprofloxacin is depicted in the structure as red while GSK299423 is green. Resistance determinants for EN-7 (blue), ciprofloxacin (yellow), and GSK299423 (teal) are also depicted.

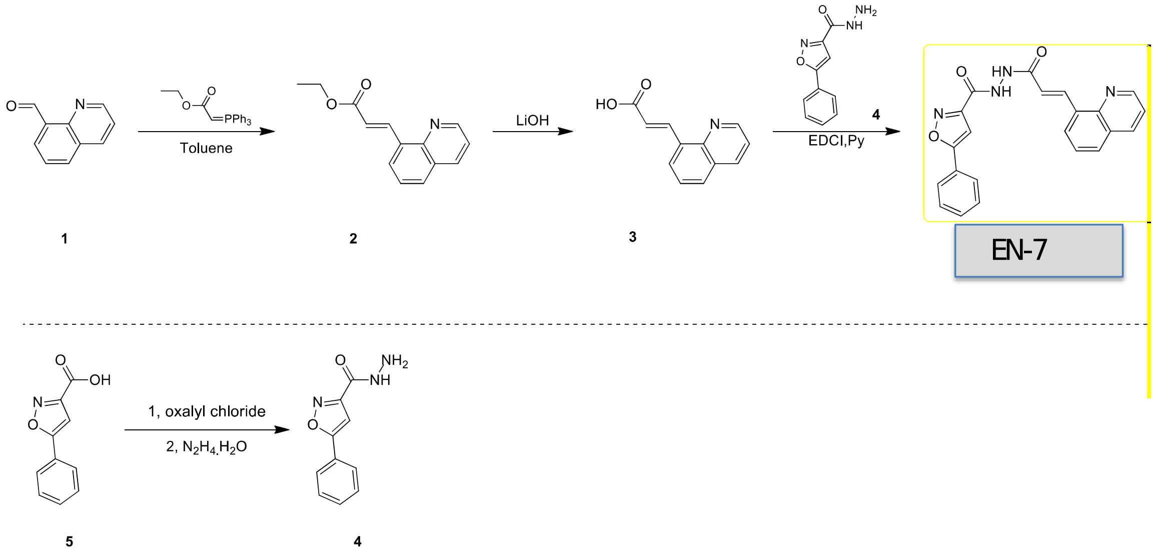

Figure S5, related to STAR Method, Synthesis and characterization of EN-7

Strain	C0117	C0023	C0112	C0017	C0032	C0018	C0019	C0024		
MIC (μM)	> 128	1	4	1	> 128	> 128	1	1		
**Resistance elements**									**Product**	**Inhibited antibiotics**
parE P585S	■						■		Modified topoisomerase IV	No reference
ErmA	■	■	■	■					Ribosomal RNA methyltransferase	Macrolides
ANT(4')-Ib	■	■	■	■					Nucleotidyltransferase	Aminoglyclosides
ANT(9)-Ia	■	■	■	■					Nucleotidyltransferase	Aminoglyclosides
blaZ			■	■	■	■	■		Beta-lactamases	Beta-lactams
SAT-4					■	■			Acetyltransferase	Nucleosides
mphC					■	■			Phosphotransferase	Macrolides
APH(3')-IIIa					■	■			Phosphotransferase	Macrolides
bcrA					■	■			ABC efflux pump	Bacitracin
gyrB K66Q					■	■	■	■	Modified gyrase	No reference
gyrA R246C			■						Modified gyrase	No reference
gyrA T457A		■				■	■	■	Modified gyrase	No reference
gyrA D459N		■				■	■	■	Modified gyrase	No reference
gyrA S85P		■		■					Modified gyrase	Fluoroquinolones
gyrA S84L	■	■	■	■	■	■	■	■	Modified gyrase	Fluoroquinolones
mecI	■	■	■	■	■	■	■		PBP2A	Beta-lactams
mprF	■	■	■	■	■	■	■	■	Modified phosphatidylglycerol	Daptomycin
tet38	■	■	■	■	■	■	■	■	MFS efflux pump	Tetracycline
Sav1866	■	■	■	■	■	■	■	■	ABC efflux pump	Multidrug
norB	■	■	■	■	■	■	■	■	MFS efflux pump	Fluoroquinolones & Tetracycline
norA	■	■	■	■	■	■	■	■	MFS efflux pump	Fluoroquinolones & Tetracycline
murA	■	■	■	■	■	■	■	■	UDP-N-acetylglucosamine enolpyruvyl transferase	Fosfomycin
mgrA	■	■	■	■	■	■	■	■	MFS efflux pump	Multidrug
mepR	■	■	■	■	■	■	■	■	MATE transporter	Tetracycline
mepA	■	■	■	■	■	■	■	■	MATE transporter	Tetracycline
mecR1	■	■	■	■	■	■	■	■	PBP2A	Beta-lactams
mecC	■	■	■	■	■	■	■	■	PBP2A	Beta-lactams
lmrB	■	■	■	■	■	■	■	■	ABC efflux pump	Lincosamides
EF-Tu	■	■	■	■	■	■	■	■	Elongation factor Tu	Kirromycin
cls	■	■	■	■	■	■	■	■	Cardiolipin synthetase	Daptomycin
dfrC	■	■	■	■	■	■	■	■	Dihydrofolate reductase	Trimethoprim
ileS	■	■	■	■	■	■	■	■	Isoleucyl-tRNA synthetase	Mupirocin
arlS	■	■	■	■	■	■	■	■	MFS efflux pump	Fluoroquinolones
alaS	■	■	■	■	■	■	■	■	Alanyl-tRNA synthetase	Novobiocin
arlR	■	■	■	■	■	■	■	■	MFS efflux pump	Fluoroquinolones

Table S1, related to Table 1. EN-7 inhibits growth of extensively resistant *S. aureus* clinical strains. Blue boxes indicates that the corresponding antibiotic resistance gene was found within the clinical strain's genome. A white box indicates that the gene was absent in the genome.
