# Supplementary material for: Discovery of a Novel DNA Gyrase-Targeting Antibiotic through the Chemical Perturbation of Streptomyces venezuelae Sporulation
Source: Cell Chem Biol. 2019 Sep 19;26(9):1274–1282.e4. doi: 10.1016/j.chembiol.2019.06.002 (PMC6856721; doi:10.1016/j.chembiol.2019.06.002)
Supplement: Document S2. Article plus Supplemental Information [file mmc2.pdf]

Article

Cell Chemical Biology

Discovery of a Novel DNA Gyrase-Targeting Antibiotic through the Chemical Perturbation of *Streptomyces venezuelae* Sporulation

Graphical Abstract

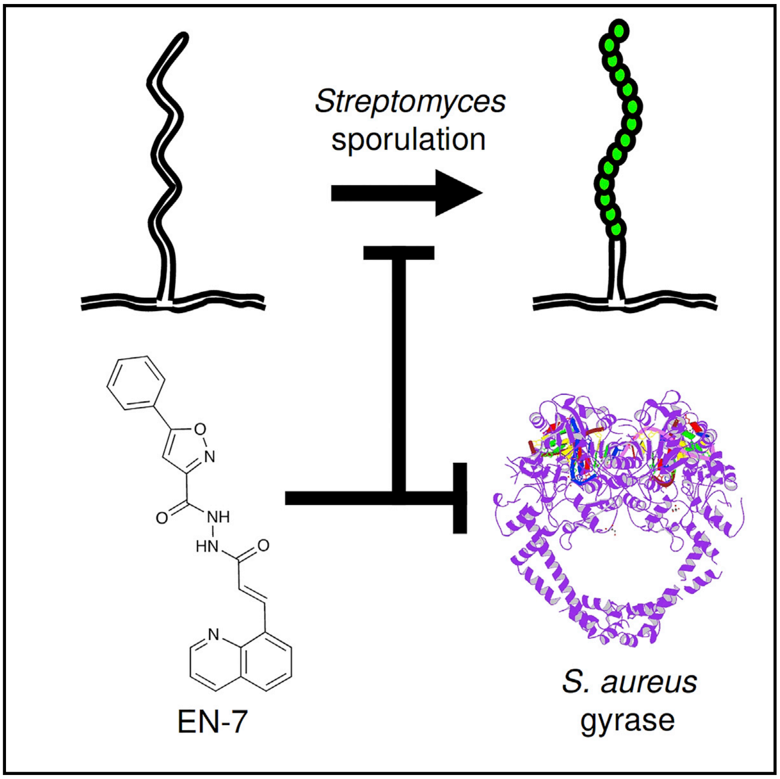

Highlights

- *Streptomyces* sporulation is sensitive to chemically induced DNA damage
- Screening 3,705 synthetic molecules uncovered novel sporulation inhibitors
- Lead molecule, EN-7, is an inhibitor of extensively resistant Gram-positive pathogens
- EN-7 targets DNA gyrase

Authors

Scott McAuley, Alan Huynh, Alison Howells, Chris Walpole, Anthony Maxwell, Justin R. Nodwell

Correspondence

justin.nodwell@utoronto.ca

In Brief

Streptomyces sporulation is the final stage in the bacteria's multicellular life cycle. McAuley and colleagues show that this process is highly sensitive to small-molecule-induced DNA damage, and use this effect to identify EN-7, a small-molecule inhibitor of bacterial gyrase.

McAuley et al., 2019, Cell Chemical Biology *26*, 1274–1282
September 19, 2019 © 2019 The Author(s). Published by Elsevier Ltd.
https://doi.org/10.1016/j.chembiol.2019.06.002

CellPress

Discovery of a Novel DNA Gyrase-Targeting Antibiotic through the Chemical Perturbation of *Streptomyces venezuelae* Sporulation

Scott McAuley,[1] Alan Huynh,[1] Alison Howells,[2] Chris Walpole,[3] Anthony Maxwell,[4] and Justin R. Nodwell[1,5,*]
[1]Department of Biochemistry, University of Toronto, Toronto, ON M5G 1M1, Canada
[2]Inspiralis Ltd., Innovation Centre, Norwich Research Park, Colney Lane, Norwich NR4 7UH, UK
[3]Structural Genomics Consortium, Research Institute of the McGill University Health Centre, Montreal, QC H4A 3J1, Canada
[4]Department of Biological Chemistry, John Innes Centre, Norwich Research Park, Norwich NR4 7UH, UK
[5]Lead Contact
*Correspondence: justin.nodwell@utoronto.ca
https://doi.org/10.1016/j.chembiol.2019.06.002

SUMMARY

Common approaches to antibiotic discovery include small-molecule screens for growth inhibition in target pathogens and screens for inhibitors of purified enzymes. These approaches have a shared intent of seeking to directly target a vital Achilles heel in a pathogen of interest. Here, we report the first screen against a sporulation pathway in a non-pathogenic bacterium as a means of discovering novel antibiotics—this effort has resulted in two important discoveries. First, we show that the sporulation program of *Streptomyces venezuelae* is exquisitely sensitive to numerous forms of DNA damage. Second, we have identified a DNA gyrase inhibitor. This molecule, EN-7, is active against pathogenic species that are resistant to ciprofloxacin and other clinically important antibiotics. We suggest that this strategy could be applied to other morphogenetic pathways in prokaryotes or eukaryotes as a means of identifying novel chemical matter having scientific and clinical utility.

INTRODUCTION

It is widely acknowledged that there is a significant and unmet need for new antibacterial compounds because of the growing and global problem of antibiotic resistance. At present, significant discovery efforts in this area are focused on the chemical evolution of existing antibiotic classes to circumvent resistance (Fernandes and Martens, 2017). Alternatively, searches for new compounds are routinely initiated with high-throughput screens of vast compound libraries to identify chemical matter that specifically inhibits the growth of target pathogens (Zlitni et al., 2009) or directly inhibits a particular resistance mechanism (Tehrani and Martin, 2018). Screens against specific molecular targets, reconstituted *in vitro*, have also served to provide lead compounds for antibiotic development (Eakin et al., 2012). All these approaches have merits; however, given the lack of new antibacterial scaffolds in clinical trials, it is clear that new thinking is also needed. For example, compounds that interfere with specific metabolic states (Gehrke et al., 2017), previously untargeted molecular processes (Haydon et al., 2008), or bacterial morphology (Bean et al., 2009) also have significant potential for antibiotic discovery. We have taken a unique approach by screening against the sporulation pathway in the bacterial genus *Streptomyces.*

The streptomycetes are filamentous bacteria that exhibit a complex multicellular life cycle. This life cycle starts with spore germination, continues with the growth of vegetative "substrate hyphae," followed by the erection of a fuzzy layer of "aerial hyphae" on the colony surface. The aerial hyphae then undergo a concerted round of cell division that divides each filament into a chain of unigenomic compartments that then mature into spores (McCormick and Flärdh, 2012). In contrast, the substrate hyphae are sites of "secondary metabolism" that generate antibacterial compounds, which, in nature, are believed to protect the developing colony from competing microorganisms (Bibb, 1996). Each stage of this process is accompanied by a visual phenotype: defects in spore maturation manifest as "white" mutants because the colonies are unable to synthesize the WhiE spore pigment that is believed to be the final step in this life cycle (Davis and Chater, 1990).

In previous research, we have screened compound libraries for molecules that upregulate secondary metabolism in *Streptomyces coelicolor* (Craney et al., 2012; Pimentel-Elardo et al., 2015). We noted in this and subsequent work that some of the compounds we discovered appeared to have effects on other aspects of the streptomycetes life cycle, including sporulation (Ahmed et al., 2013). Intriguingly, some of the compounds that target sporulation turned out to have antibacterial effects against rod-shaped and coccoid bacteria, such as *Bacillus subtilis* and *Staphylococcus aureus.* These molecules targeted cells in unusual ways that are clearly outside the most common suite of antibiotic targets: one compound blocked septation (Jani et al., 2015) and another interfered with the relationship between cell growth and cell division (McAuley et al., 2019).

Having discovered that some inhibitors of the *Streptomyces* sporulation pathway have antibiotic activity against rod-shaped and coccoid bacteria, we decided to determine whether the opposite was also true. By screening antibiotics having known

 © 2019 The Author(s). Published by Elsevier Ltd.
This is an open access article under the CC BY license (http://creativecommons.org/licenses/by/4.0/).

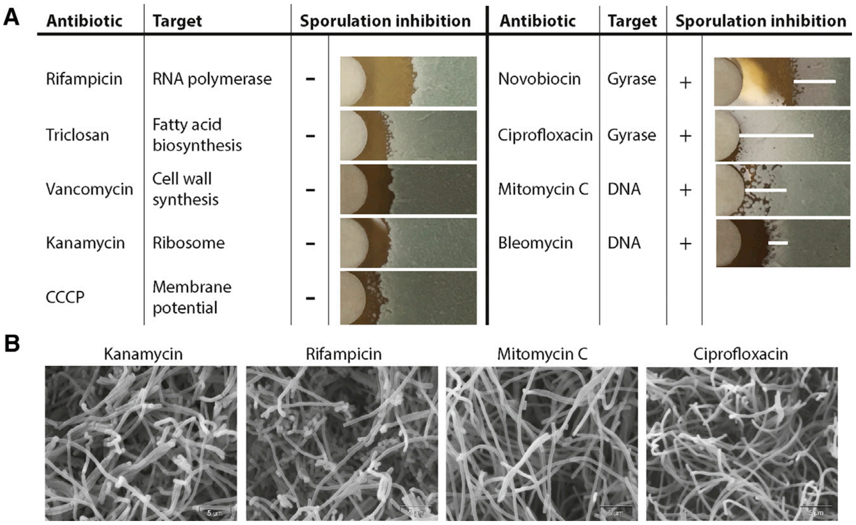

Figure 1. *S. venezuelae* Sporulation Is Sensitive to DNA Damage

(A) *S. venezuelae* is treated with antibiotics of various targets to determine the impact on growth development. Following 48 h of incubation sporulation inhibition is visualized by the appearance of a white halo (*whi* phenotype) in the sub-MIC region surrounding the zone of inhibition. A white horizontal line has been added to the sporulation-deficient panes as a visual guide. Each disk is treated with 10 μL of the following antibiotics, 100 μg/mL rifampicin, 1 mg/mL triclosan, 100 μg/mL vancomycin, 250 μg/mL kanamycin, 3 mM CCCP, 100 μg/mL novobiocin, 250 μg/mL ciprofloxacin, 20 μg/mL mitomycin C, and 100 μg/mL bleomycin. The concentrations were selected in order to get a relatively consistent zone of inhibition between the various treatment molecules.

(B) Scanning electron microscopy images near the zone of inhibition of *S. venezuelae* treated with kanamycin, rifampicin, mitomycin C, and ciprofloxacin.

targets we report that compounds targeting the integrity of DNA confers a sporulation defect at sub-inhibitory concentrations, whereas compounds that target the ribosome, RNA polymerase, the cell wall, and the cell's electrochemical gradient have only a simple antibacterial effect. We leveraged this discovery by screening a small library of 3,705 synthetic compounds, previously demonstrated to have biological activity against Gram-positive bacteria (Czarny and Brown, 2016; McAuley et al., 2018), for compounds having the capacity to inhibit sporulation in *Streptomyces venezuelae.* Here we report that the most potent of these is an inhibitor of DNA gyrase identified as EN-7.

RESULTS

The *Streptomyces venezuelae* Sporulation Program Is Sensitive to DNA Damage

To determine the effect of chemical inhibitors on morphogenesis in *S. venezuelae*, we applied antibiotics having diverse molecular targets at concentrations 5–10× their minimum inhibitory concentration (MIC) to filter disks on lawns of spores. We then observed how these molecules impacted growth and development (Figure 1A). Antibiotics such as rifampicin, triclosan, vancomycin, and kanamycin conferred a zone of inhibited growth as expected. Growth and sporulation were normal at concentrations below the MIC, as demonstrated by the green pigmentation of the lawn surface. This was also the effect of the ionophore carbonyl cyanide m-chlorophenyl hydrazone (CCCP). In contrast, compounds that either directly target DNA or inhibit bacterial gyrase conferred a white phenotype at sub-inhibitory concentrations, consistent with a failure to produce the WhiE spore pigment associated with the completion of the *S. venezuelae* sporulation cycle (Santos-Beneit et al., 2017; Santos-Beneit et al., 2017b).

To confirm that DNA damage blocked sporulation we carried out scanning electron microscopy (SEM) on a section of the *S. venezuelae* lawn immediately adjacent to the zone of inhibition formed by treatment with mitomycin C (DNA crosslinker), ciprofloxacin (gyrase inhibitor), kanamycin (translation inhibitor), and rifampicin (transcription inhibitor) (Figure 1B). We found that, while the kanamycin and rifampicin images show septated aerial hyphae, indicating sporulation, the samples treated with the DNA damage agents mitomycin C and ciprofloxacin do not show any aerial hyphae septation. This confirms that the white phenotype induced by these molecules is caused by an inhibition of *S. venezuelae* sporulation septation.

Screening for Novel Small-Molecule Sporulation Inhibitors

To determine whether this phenomenon could be used to identify novel antimicrobial agents, we screened 3,705 synthetic bioactive small molecules (Czarny and Brown, 2016) via a disk diffusion assay for the capacity to block sporulation in *S. venezuelae* at sub-MIC concentrations. In this way, we identified ten compounds, all of which had antibacterial activity at higher concentrations but that conferred a reproducible white phenotype below the MIC (Figure S1).

Combining the effect on both growth and sporulation inhibition, the most potent of these molecules was EN-7 (Figure 2A). Treatment with a filter disk containing 10 μL of 2.5 mM EN-7 resulted in a significant zone of inhibition as well as a striking band of inhibited sporulation below the MIC for growth (Figure 2B). To confirm that the white phenotype in this zone was due to inhibited sporulation, green and white sections of the lawn were imaged by SEM. Consistent with our screening criteria, the green region showed a healthy lawn containing aerial hyphae septated into mature spores while the white region showed aerial hyphae without any septation. In spite of the exceptional EN-7 sensitivity of the *S. venezuelae* sporulation program, the compound conferred no other morphological defects.

To determine the molecule's effect on non-streptomycetes we tested EN-7 against a series of three *S. aureus* and three *Escherichia coli* laboratory strains, as well as eight extensively resistant clinical *S. aureus* strains (Table 1). For the Gram-positive *S. aureus*, we found that the molecule had an MIC of 1 μM, or 0.4 μg/mL, against the three laboratory strains (ATCC 29213, TCH1516, and ATCC 33591) and four clinical strains (C0017, C0024, C0023, and C0019), an increased MIC of 4 μM against one of the clinical strains (C0112) and was inactive against the

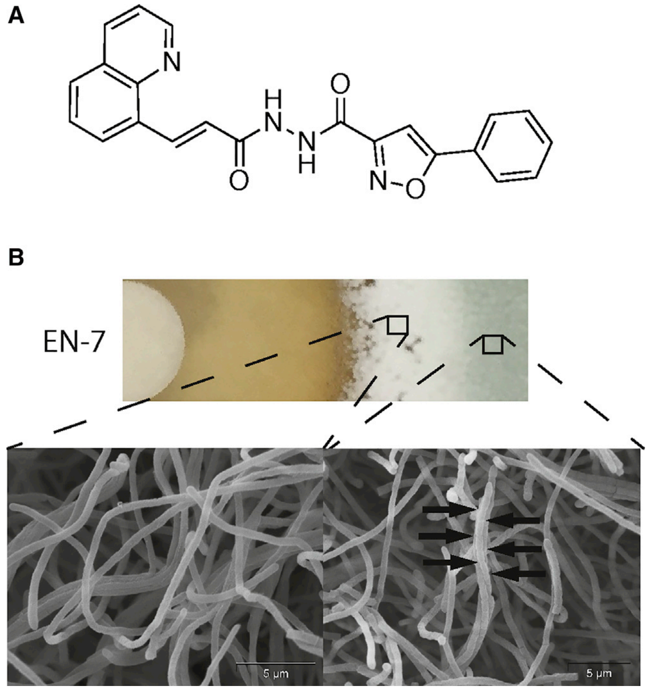

Figure 2. EN-7 Inhibits *S. venezuelae* Growth and Sporulation

(A) Chemical structure of EN-7.

(B) Sporulation inhibition phenotype induced by EN-7. A section of the lawn was cut from the agar and both the treated (left) and untreated (right) sections imaged with scanning electron microscopy.

Table 1. MIC of EN-7 against Lab and Clinical Strains of *S. aureus* and *E. coli*

Species	Strain	MIC (µM)
S. aureus	ATCC 29213	1
	TCH 1516	1
	ATCC 33519	1
	C0117	>128
	C0023	1
	C0112	4
	C0017	1
	C0032	>128
	C0018	>128
	C0019	1
	C0024	1
E. coli	MC1061	>64
	BW25113	>64
	Δ*tolC* Δ*bamB*	1

other three clinical strains (C0018, C0032, and C0117) at concentrations up to 128 µM. The antibiograms for these clinical strains indicated extensive antibiotic resistance to ciprofloxacin, clindamycin, erythromycin, levofloxacin, and oxacillin. In addition, the Comprehensive Antibiotic Resistance Database (Jia et al., 2017) was used to identify many of the antibiotic resistance genes or modified targets within the genomes of these strains (Table S1). While we could not find a definitive explanation for the difference in activity, it is clear that EN-7 is active against clinically relevant strains and can overcome a diverse range of resistance mechanisms. Against the Gram-negative *E. coli*, we observed no growth inhibition against the MC1061 (Casadaban and Cohen, 1980) or BW25113 (Datsenko and Wanner, 2000) strains at concentrations up to 64 µM but did observe an MIC of 1 µM against the outer-membrane and efflux-deficient *E. coli* BW25113 Δ*tolC* Δ*bamB* strain (King et al., 2014). Thus, EN-7 is highly active against even extensively resistant *S. aureus*, and the resistance observed in Gram-negative *E. coli* is likely caused by an inability to enter the cell through the outer-membrane.

Since EN-7 is a hit from a synthetic small-molecule library, we wanted to confirm the molecule's structure to ensure the observed activity was not caused by a degradation product. The molecule was synthesized (Figure S5) and confirmed that it has the same activity as the hit molecule from the library.

EN-7 Targets DNA Gyrase

To understand EN-7's mechanism of action, we raised nine EN-7^R mutants from *S. aureus* strain ATCC 29213. We isolated three such mutants using a serial passaging approach (mut-1, mut-2, and mut-3) and six more by direct plating on agar containing 10 µM, or 10× MIC, of EN-7 (mut-4 through mut-9).

We determined full-length chromosome sequences of the nine EN-7-resistant strains and found that each of them had acquired a point mutation in the *gyrAB* operon (Table 2). Three of the mutants had an altered GyrA sequence: A34T (mut-3 and mut-9) and P219Q (mut-6). The other six mutants had an altered GyrB sequence: K417E (mut-1, mut-2, mut-4, and mut-5) and D437N (mut-7 and mut-8). We confirmed the EN-7 resistance of these cultures using a broth dilution MIC assay, showing no activity up to 16 µM (16× MIC), the highest concentration tested (Figure 5).

To confirm that the mutations are the cause of the observed EN-7 resistance we selected the most common mutation, GyrB K417E, and reconstituted it in wild-type *S. aureus* ATCC 29213 through allelic exchange (Monk et al., 2012). Because of the essential nature of gyrase, the allelic exchange was accomplished in a single step by spreading plasmid-containing cells as well as wild-type *S. aureus* ATCC 29213 cells to agar plates containing 10 µM EN-7 for final selection and 1 µg/mL anhydrotetracycline to activate the pIMAY anti-*secY* sequence and ensure the plasmid itself was not integrated into the chromosome. The plates were then incubated overnight at the plasmid non-replicating temperature of 37°C. Following overnight incubation, we observed approximately 50 colonies on each of the wild-type plates and over 2,000 on each of the plates containing the GyrB K417E allelic exchange strain (Figure 3A). We confirmed the loss of the plasmid by replicate plating with 10 µM EN-7 or 10 µM EN-7 with 25 µg/mL chloramphenicol, the antibiotic selection marker for the pIMAY plasmid, and observed no growth in the presence of chloramphenicol (Figure 3B). We tested three strains in a broth dilution assay and found that they were highly resistant to EN-7, up to 128 µM (Figure 3C). Finally, we confirmed the presence of the K417E mutation by amplifying and sequencing *gyrA* and *gyrB* from eight independent allelic exchange cultures (Figure 3D). Therefore, the gyrase mutations in the EN-7^R stains are sufficient to induce EN-7 resistance.

Table 2. Summary of *S. aureus* EN-7-Resistant Mutant Strains and Corresponding Alleles

Mutant Allele	*S. aureus* EN-7^R Strains
GyrA A34T	mut-3, mut-9
GyrA P219Q	mut-6
GyrB K417E	mut-1, mut-2, mut-4, mut-5
GyrB D437N	mut-7, mut-8

Gyrase and topoisomerase IV are both type IIA topoisomerases. DNA gyrase forms double-stranded breaks and reseals DNA to induce negative supercoiling, relieving torsional stress induced by transcription or replication, as well as relaxing positive supercoiling (Vos et al., 2011). It is a tetramer composed of GyrA and GyrB (A_2B_2) that cleaves DNA through an active-site tyrosine located on each GyrA subunit, is highly conserved across bacteria, and its function can be inhibited through multiple mechanisms and by several different antibiotic classes. Topoisomerase IV, while also able to cleave and reseal double-stranded DNA and composed of a heterotetramer of ParC and ParE (C_2E_2), has a different function than gyrase. It is responsible for decatenating DNA following replication and relaxing positive DNA supercoils (Blanche et al., 1996; Hooper and Jacoby, 2016).

To directly test EN-7 activity, we utilized a series of *in vitro* assays against purified *S. aureus* and *E. coli* gyrase and topoisomerase IV enzymes (Figure 4). First, we found that EN-7 inhibits *S. aureus* gyrase supercoiling activity with a half maximal inhibitory concentration (IC_{50}) of 85 nM (Figure 4A) while inhibiting *E. coli* gyrase supercoiling at an IC_{50} of 1.75 μM (Figure 4B). The activity against topoisomerase IV-catalyzed decatenation was reversed, with inhibition of *E. coli t*opoisomerase IV at an IC_{50} of 0.89 μM (Figure 4C) with no activity against the *S. aureus* enzyme up to 100 μM (Figure 4D). To investigate possible mechanisms of gyrase inhibition, we found that EN-7 does not stabilize the gyrase-DNA double-stranded cleavage complex in a manner similar to fluoroquinolones but does increase the occurrence of DNA nicking (Figure 4E). Co-treatment with both ciprofloxacin and EN-7 does not prevent ciprofloxacin-induced formation of the double-stranded cleavage complex (Figure 4F), suggesting that EN-7 does not inhibit gyrase-DNA binding and acts in a manner that does not prevent the formation of the double-stranded break. In addition, EN-7 inhibits *S. aureus* gyrase-induced relaxation at an IC_{50} of 0.1–0.5 μM (Figure 4G). Since this process acts independently of ATPase activity, this inhibition confirms that the molecule does not act as an ATPase inhibitor. Finally, we found that *S. aureus* gyrase containing the fluoroquinolone resistance mutation GyrA S84L is inhibited by EN-7 at an IC_{50} of 9.96 μM, over a 100-fold increase relative to the wild-type enzyme (Figure 4H). This collection of *in vitro* assays demonstrates that EN-7 is a highly potent topoisomerase inhibitor that acts independently of conventional mechanisms while being less active, *in vitro*, against gyrase containing the GyrA S84L mutation.

We took two approaches to determine whether the EN-7^R mutations fall in previously identified functional regions of the DNA gyrase enzyme. First, we mapped them into an alignment of published GyrA and GyrB sequences from *S. aureus*, *E. faecalis*, *S. coelicolor*, *S. venezuelae*, *B. subtilis*, and *E. coli* (Figure S4A). All four of the residues altered in EN-7^R strains are fully conserved across these six diverse bacteria. Second, we mapped our EN-7-resistant mutations to published gyrase structures in complex with ciprofloxacin (2XCT) and GSK299423 (2XCS), a novel bacterial topoisomerase inhibitor (NBTI) molecule and compared them to reported resistance mutations (Figures S4B and S4C) (Bax et al., 2010). Although the mutations do not cluster around a specific binding site, all four of the mutations are distinct from common fluoroquinolone resistance mutations at GyrA S84, S85, and E88 (Aldred et al., 2013), NBTI NXL101 resistance mutation GyrA M121K, thiophene resistance mutations GyrB E634, GyrB R630, and P343 (Chan et al., 2017), and the proposed NBTI-gyrase interaction pocket GyrA A68, G72, M75, and M121 (Bax et al., 2010). Interestingly, two of our EN-7^R mutations have been reported previously. GyrB D437N results in resistance to QPT-1, etoposide, and AZD0914 (Chan et al., 2015) as well as the spiropyrimidinetrione ETX0914 (Basarab et al., 2015), while both GyrB D437N and GyrB K417E mutations result in resistance to NBTI's AZ6142, AZ0217, and MCHEM18 (Lahiri et al., 2015). In addition, a nearby GyrA A32V mutation has been shown to alter sensitivity to these same NBTI molecules (Lahiri et al., 2015). Structurally, both the GyrB D437N and GyrB K417E mutations are located in the DNA binding TOPRIM domain and directly contact the DNA backbone. The GyrA A34T mutation is found in the winged helix DNA binding domain and interacts with the DNA backbone far from the NBTI binding site, while P219Q is located further from the direct DNA binding site.

To investigate any biological cross-resistance to other gyrase inhibitors we tested the EN-7^R strains against ciprofloxacin, novobiocin, and gepotidacin (Figure 5). We observed no significant cross-resistance with ciprofloxacin or novobiocin. The MICs of ciprofloxacin and novobiocin against the mutants were the same as those against the parent strain, 0.2 and 0.06 μg/mL respectively. However, we observed partial cross-resistance with gepotidacin. The NBTI molecule inhibited growth of the wild-type strain at 0.5 μM. Strains containing GyrA A34T (mut-3 and mut-9), GyrA P219Q (mut-6), and GyrB K417E (mut-1, mut-2, mut-4, and mut-5) exhibited moderate resistance, with MICs of either 4 or 8 μM, while the strains containing GyrB D437N (mut-7 and mut-8) displayed a higher level of resistance with an MIC of 32 μM. This suggests that EN-7 may share resistance elements with gepotidacin.

DISCUSSION

In this study, we describe the discovery of a gyrase inhibitor, EN-7, from a screen against *Streptomyces* sporulation septation. We have previously used *Streptomyces* as a small-molecule screening platform for compounds that affect secondary metabolism (Craney et al., 2012) and sporulation (Jani et al., 2015), which is a form of developmentally controlled cell division. *Streptomyces* sporulation employs specific genes that drive changes in cell morphology and fate. However, these sporulation mechanisms also co-opt core macromolecular machinery traditionally employed for vegetative growth and division, exposing unique points of vulnerability. Here, using the classic white (*whi*) phenotype to identify sporulation inhibition, we identify that *S. venezuelae* sporulation is highly sensitive to

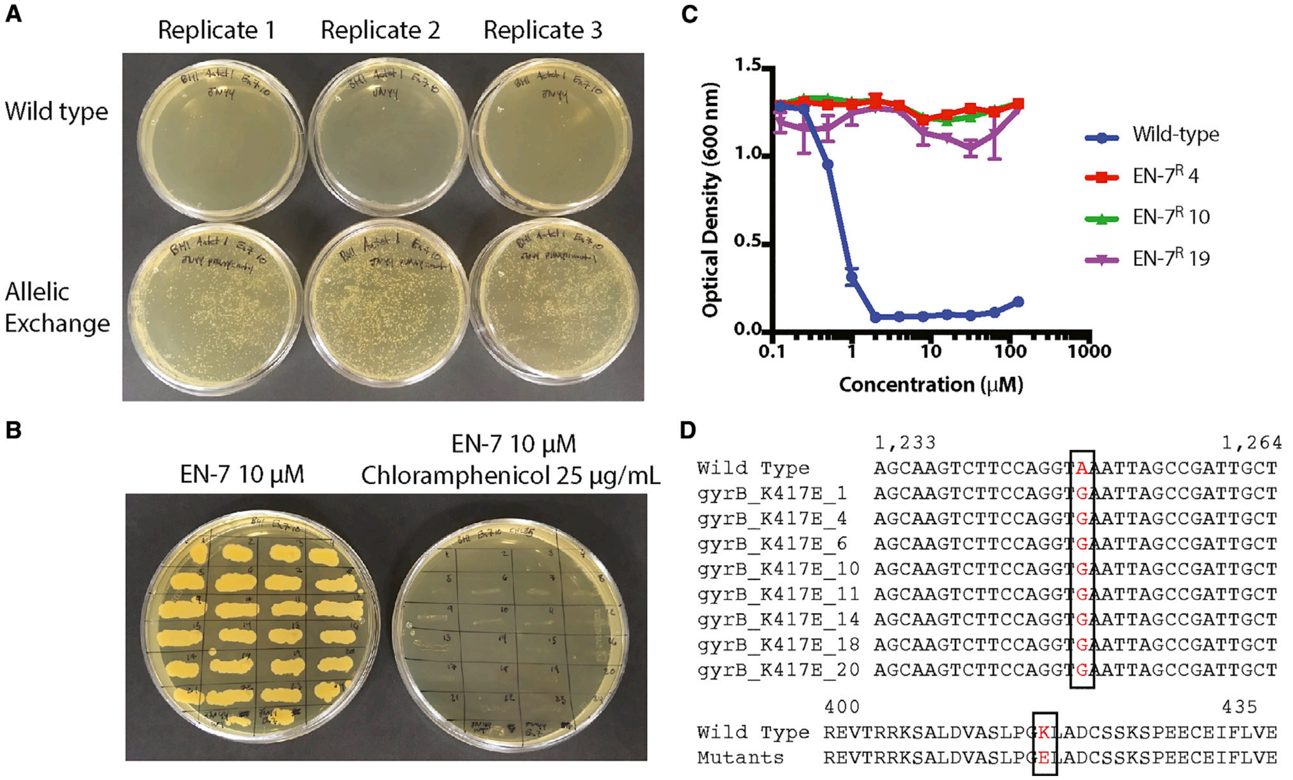

Figure 3. Allelic Exchange of EN-7 Resistance Mutations into *S. aureus*

(A) Image of three replicate plates of the allelic exchange of the EN-7 resistance GyrB K417E mutation into *S. aureus* versus wild-type *S. aureus* following incubation in the presence of 1 μg/mL anhydrotetracycline and 10 μM EN-7 and incubated at the pIMAY non-replicating temperature of 37°C.

(B) Replicate plating of 24 colonies from the allelic exchange plates in (A) to agar containing 10 μM EN-7 (left) and 10 μM EN-7 and 25 μg/mL chloramphenicol (right) demonstrating the loss of the pIMAY plasmid following allelic exchange.

(C) Determining MIC of three of the replicate plated EN-7-resistant strains from (B) showing no EN-7 activity up to 128 μM relative to the MIC of 1 μM in the wild-type *S. aureus* ATCC 29213 strain.

(D) Sequencing of the *gyrB* amplicon showing the point mutation in each of the tested allelic exchange strains (top) and the translation of the corresponding region showing the K417E amino acid substitution (bottom).

known antibiotics that directly target DNA or disrupt bacterial gyrase. This is the first report that such diverse forms of DNA damage block sporulation in a streptomycete.

While DNA damage-induced cell division inhibition has been extensively described in other bacteria, such a system is not known within *Streptomyces.* Across diverse bacteria, the SOS response induces the expression of specific proteins that delay cell division. This includes YneA in *B. subtilis* (Kawai et al., 2003), SulA in *E. coli* (Bi and Lutkenhaus, 1993), SosA in *S. aureus* (Bojer et al., 2018), Rv2719c in *Mycobacterium* (Chauhan et al., 2006), and DivS in *Corynebacterium* (Ogino et al., 2008). Although *S. venezuelae* contains homologs to the SOS-response modulators LexA and RecA, no equivalent division inhibitor has yet been identified. However, our findings demonstrate that *S. venezuelae* is very sensitive to a variety of forms of DNA damage and responds by delaying sporulation, likely to prevent passing damaged chromosomes to its progeny.

We then used this effect as a platform for screening small molecules for sporulation inhibition and identified ten active molecules. The most potent of these across both growth inhibition and sporulation inhibition is EN-7, a bacterial topoisomerase inhibitor. EN-7 is highly active against Gram-positive pathogens, including extensively resistant clinical *S. aureus* strains containing resistant elements to fluoroquinolones and numerous other antibiotic classes. Although we did not observe activity against wild-type *E. coli*, an efflux and outer-membrane-deficient strain shows comparable activity with *S. aureus*, suggesting either that the outer-membrane is inhibiting entry of the molecule to the cell or that the compound is being removed from the cell by efflux. We also found no toxicity to human HEK293 cells (Figure S3).

Both genetic and biochemical methods were used to identify DNA gyrase as the target of EN-7. We observe that, in *S. aureus*, EN-7 strongly inhibits gyrase-induced negative supercoiling while having no impact on topoisomerase IV decatenation activity. However, in *E. coli* the molecule inhibits both enzymes while maintaining greater activity against gyrase. This difference in activity across these enzymes in different bacteria can be seen in other topoisomerase inhibitors. For example, ciprofloxacin is more potent against *S. aureus* topoisomerase IV than gyrase (Blanche et al., 1996), norfloxacin preferentially targets topoisomerase IV, and nalidixic acid targets gyrase (Fournier et al., 2000), whereas in *E. coli*, ciprofloxacin preferentially inhibits gyrase over topoisomerase IV (Khodursky et al., 1995). Therefore, the strong preference for gyrase inhibition in

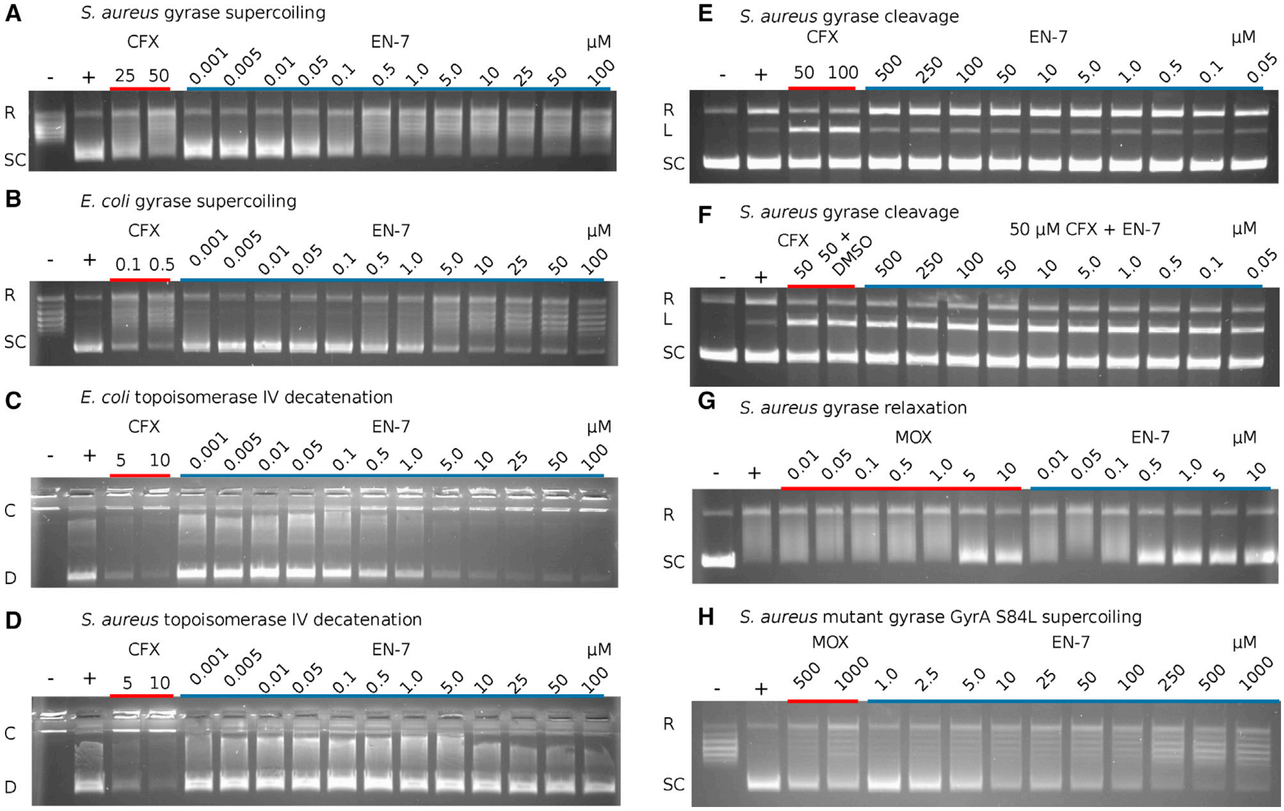

Figure 4. ***In Vitro*** **Inhibition of Topoisomerase Function by EN-7**

pBR322 DNA was incubated with gyrase or topoisomerase IV and different concentrations of compound and separated by agarose gel electrophoresis. (+) is a positive control with untreated enzyme, while (−) is a no-enzyme control. Labels indicate relaxed (R), supercoiled (SC), linear (L), catenated (C), and decatenated (D) DNA.

(A) Inhibition of *S. aureus* gyrase supercoiling by EN-7.

(B) Inhibition of *E. coli* gyrase supercoiling by EN-7.

(C) Inhibition of *E. coli* topoisomerase IV decatenation by EN-7.

(D) Inhibition of *S. aureus* topoisomerase IV decatenation by EN-7.

(E) Unlike ciprofloxacin (CFX), EN-7 does not stabilize a double-stranded break DNA gyrase cleavage complex with *S. aureus* gyrase.

(F) EN-7 does not inhibit the formation of ciprofloxacin (CFX) stabilized cleavage complex.

(G) EN-7 and moxifloxacin (MOX) inhibit ATP-independent *S. aureus* gyrase-induced DNA relaxation.

(H) The *S. aureus* gyrase mutant GyrA S84L is less susceptible to both MOX and EN-7.

both Gram-positive and Gram-negative versions of the enzyme distinguishes EN-7 from its traditional counterparts.

Our data show EN-7 to be distinct from classical topoisomerase inhibitors. Our EN-7-resistant *S. aureus* strains do not demonstrate cross-resistance to either ciprofloxacin or novobiocin, and EN-7 is active against ciprofloxacin-resistant clinical *S. aureus* strains that, among other elements, contain the GyrA S84L ciprofloxacin resistance mutation. Unlike ciprofloxacin, EN-7 does not stabilize a double-stranded DNA gyrase cleavage complex and, unlike novobioxin, does not interfere with the ATPase function of gyrase. Despite this significant whole-cell activity, purified *S. aureus* gyrase containing the GyrA S84L mutation shows ~100-fold resistance to EN-7-induced supercoiling inhibition. Combined with the molecule's ability to inhibit gyrase-induced DNA relaxation, this highlights that the biological activity of EN-7 extends beyond inhibiting supercoiling.

There are a growing number of gyrase inhibitors under investigation, including simocylinone D8 (Buttner et al., 2018), thiophenes (Chan et al., 2017), imidazopyrazinones (Germe et al., 2018), and the NBTIs (Bax et al., 2010), such as gepotidacin (Biedenbach et al., 2016; Flamm et al., 2017; Taylor et al., 2018; Gibson et al., 2019). These inhibitors act by inhibiting DNA gyrase binding, stabilizing a pre-cleaved complex, or stabilizing DNA cleavage complexes, highlighting the diverse mechanisms of gyrase inhibition. While EN-7 is structurally unique, it shares some superficial structural features with NBTIs, including a quinoline group connected to a heterocycle via a flexible linker region (Figure S2). However, our *in vitro* results show that EN-7 does not act via the same mechanism as these newer gyrase inhibitors. EN-7 does not inhibit DNA gyrase binding, such as simocyclinone D8; does not prevent the formation of fluoroquinolone-induced double-stranded breaks during co-treatment, such as gepotidacin; and does not stabilize a gyrase-DNA cleavage complex, such as imidazopyrazinones and thiophenes. Therefore, EN-7 inhibits topoisomerase activity via a mechanism that is independent from existing molecules.

Beginning with a screen for chemical inhibitors of *S. venezuelae* sporulation we have uncovered a gyrase inhibitor. EN-7 inhibits

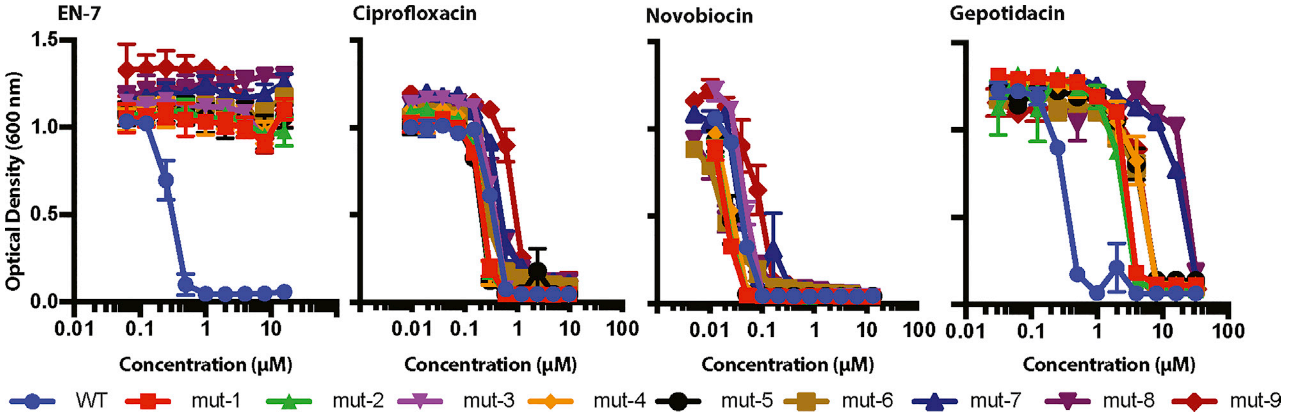

Figure 5. Cross-Resistance of EN-7^R Strains with Other Gyrase Inhibitors

No observed cross-resistance between EN-7 and other gyrase-targeting antibiotics, ciprofloxacin and novobiocin; however, there is some cross-resistance to the NBTI gepotidacin.

both gyrase-induced supercoiling and relaxation, stimulates the formation of single-stranded breaks, and inhibits growth of fluoroquinolone-resistant clinical strains. EN-7 may be acting by altering the dynamics of gyrase-induced DNA cleavage and religation by stimulating the formation of single-stranded breaks. However, uncovering the precise mechanisms by which this is inhibiting DNA supercoiling and bacterial growth will require additional structural and biochemical study.

Finally, we suggest that there are many pathways that could be subjected to similar screening methods, including endospore formation in *Bacillus subtilis*, myxospore formation in *Myxococcus xanthus*, and even sporulation programs in eukaryotic organisms such as *Saccharomyces cerevisiae*. Chemical screens against these morphogenetic programs could be a valuable means of identifying new chemical matter of potential clinical interest.

SIGNIFICANCE

The rise of antibiotic resistance has led to a significant need for new therapeutics. Here, we describe that *Streptomyces* sporulation is highly sensitive to DNA disrupting antibiotics and how this can be harnessed to identify novel bioactive molecules. The *Streptomyces* genus are filamentous bacteria that exhibit a complex multicellular life cycle, including the growth of vegetative substrate hyphae, erection of a fuzzy layer of aerial hyphae, and a concerted round of cell division that divides each aerial filament into a chain of unigenomic compartments that then mature into spores. We show that antibiotics that directly damage DNA or disrupt DNA gyrase inhibit the developmentally regulated sporulation program. The DNA damage response in *Streptomyces* is not well understood and this finding opens new opportunities to investigate this important biological response. We also leverage this induced morphology to screen a library of small molecules to identify EN-7, a highly active molecule that inhibits bacterial gyrase. Screening against development provides additional targets and assay outputs that are not available to screens based on growth inhibition. It also provides opportunities to identify novel molecules that can inform on the underlying biology of these development programs or provide the basis of much-needed antibiotic development programs.

STAR★METHODS

Detailed methods are provided in the online version of this paper and include the following:

- KEY RESOURCES TABLE
- CONTACT FOR REAGENT AND RESOURCE SHARING
- EXPERIMENTAL MODEL AND SUBJECT DETAILS
- METHOD DETAILS
  - Strains, Media and Materials
  - Screen for Streptomyces Sporulation Inhibition
  - Scanning Electron Microscopy
  - Minimum Inhibitory Concentration
  - Isolation of EN-7 Resistant Mutants
  - Sequencing of Resistant Mutants
  - Confirming Resistance Mutations through Allelic Exchange in *S. aureus*
  - *E. coli* Gyrase Supercoiling
  - *S. aureus* Gyrase Supercoiling
  - *E. coli* Topoisomerase IV Decatenation
  - *S. aureus* Topoisomerase IV Decatenation
  - *S. aureus* Gyrase Cleavage
  - *S. aureus* Gyrase Relaxation
  - Synthesis and Characterization of EN-7
- QUANTIFICATION AND STATISTICAL ANALYSIS
- DATA AND CODE AVAILABILITY

SUPPLEMENTAL INFORMATION

Supplemental Information can be found online at https://doi.org/10.1016/j.chembiol.2019.06.002.

ACKNOWLEDGMENTS

We would like to thank Eric Brown for the *B. subtilis* active compound collection, Gerry Wright for access to the clinical strain database at the Institute for

Infectious Disease Research, Brian Coombes for the *S. aureus* TCH1516 and ATCC 33591 strains, and the Donnelly Sequencing Centre for whole-genome sequencing of the EN-7-resistant strains. We also acknowledge funding support from the Canadian Institutes of Health Research (grant MOP-133636) to J.R.N.; A.M.'s lab is supported by the Biotechnology and Biosciences Research Council (UK) Institute Strategic Program Grants BB/J004561/1 and BB/P012523/1, and the Wellcome Trust (Investigator Award 110072/Z/15/Z).

AUTHOR CONTRIBUTIONS

Conceptualization, S.McA. and J.R.N.; Methodology, S.McA.; Investigation, S.McA., C.W., A.Huynh, and A.Howells; Writing – Original Draft, S.McA.; Writing – Review & Editing, S.McA., A.M., C.W., and J.R.N.; Funding Acquisition, J.R.N.; Resources, A.M. and J.R.N.; Supervision, A.M. and J.R.N.

DECLARATION OF INTERESTS

The authors declare no competing interests.

Received: April 6, 2019
Revised: May 15, 2019
Accepted: June 13, 2019
Published: July 3, 2019

REFERENCES

Ahmed, S., Craney, A., Pimentel-Elardo, S.M., and Nodwell, J.R. (2013). A synthetic, species-specific activator of secondary metabolism and sporulation in *Streptomyces coelicolor*. Chembiochem *14*, 83–91.

Aldred, K.J., McPherson, S.A., Turnbough, C.L., Kerns, R.J., and Osheroff, N. (2013). Topoisomerase IV-quinolone interactions are mediated through a water-metal ion bridge: mechanistic basis of quinolone resistance. Nucleic Acids Res. *41*, 4628–4639.

Basarab, G.S., Kern, G.H., McNulty, J., Mueller, J.P., Lawrence, K., Vishwanathan, K., Alm, R.A., Barvian, K., Doig, P., Galullo, V., et al. (2015). Responding to the challenge of untreatable gonorrhea: ETX0914, a first-in-class agent with a distinct mechanism-of-action against bacterial type II topoisomerases. Sci. Rep. *5*, 11827.

Bax, B.D., Chan, P.F., Eggleston, D.S., Fosberry, A., Gentry, D.R., Gorrec, F., Giordano, I., Hann, M.M., Hennessy, A., Hibbs, M., et al. (2010). Type IIA topoisomerase inhibition by a new class of antibacterial agents. Nature *466*, 935–940.

Bean, G.J., Flickinger, S.T., Westler, W.M., McCully, M.E., Sept, D., Weibel, D.B., and Amann, K.J. (2009). A22 disrupts the bacterial actin cytoskeleton by directly binding and inducing a low-affinity state in MreB. Biochemistry *48*, 4852–4857.

Bi, E., and Lutkenhaus, J. (1993). Cell division inhibitors SulA and MinCD prevent formation of the FtsZ ring. J. Bacteriol. *175*, 1118–1125.

Bibb, M. (1996). 1995 Colworth Prize Lecture. The regulation of antibiotic production in *Streptomyces coelicolor* A3(2). Microbiology *142*, 1335–1344.

Biedenbach, D.J., Bouchillon, S.K., Hackel, M., Miller, L.A., Scangarella-Oman, N.E., Jakielaszek, C., and Sahm, D.F. (2016). In vitro activity of gepotidacin, a novel triazaacenaphthylene bacterial topoisomerase inhibitor, against a broad spectrum of bacterial pathogens. Antimicrob. Agents Chemother. *60*, 1918–1923.

Blanche, F., Cameron, B., Bernard, F.X., Maton, L., Manse, B., Ferrero, L., Ratet, N., Lecoq, C., Goniot, A., Bisch, D., and Crouzet, J. (1996). Differential behaviors of *Staphylococcus aureus* and *Escherichia coli* type II DNA topoisomerases. Antimicrob. Agents Chemother. *40*, 2714–2720.

Bojer, M.S., Wacnik, K., Kjelgaard, P., Gallay, C., Bottomley, A.L., Cohn, M.T., Lindahl, G., Frees, D., Veening, J.-W., Foster, S.J., et al. (2018). SosA inhibits cell division in *Staphylococcus aureus* in response to DNA damage. bioRxiv, 364299.

Buttner, M.J., Schäfer, M., Lawson, D.M., and Maxwell, A. (2018). Structural insights into simocyclinone as an antibiotic, effector ligand and substrate. FEMS Microbiol. Rev. *42*, https://doi.org/10.1093/femsre/fux055.

Casadaban, M.J., and Cohen, S.N. (1980). Analysis of gene control signals by DNA fusion and cloning in *Escherichia coli*. J. Mol. Biol. *138*, 179–207.

Chan, P.F., Germe, T., Bax, B.D., Huang, J., Thalji, R.K., Bacqué, E., Checchia, A., Chen, D., Cui, H., Ding, X., et al. (2017). Thiophene antibacterials that allosterically stabilize DNA-cleavage complexes with DNA gyrase. Proc. Natl. Acad. Sci. U S A *114*, E4492–E4500.

Chan, P.F., Srikannathasan, V., Huang, J., Cui, H., Fosberry, A.P., Gu, M., Hann, M.M., Hibbs, M., Homes, P., Ingraham, K., et al. (2015). Structural basis of DNA gyrase inhibition by antibacterial QPT-1, anticancer drug etoposide and moxifloxacin. Nat. Commun. *6*, 10048.

Chauhan, A., Lofton, H., Maloney, E., Moore, J., Fol, M., Madiraju, M.V.V.S., and Rajagopalan, M. (2006). Interference of *Mycobacterium tuberculosis* cell division by Rv2719c, a cell wall hydrolase. Mol. Microbiol. *62*, 132–147.

Craney, A., Ozimok, C., Pimentel-Elardo, S.M., Capretta, A., and Nodwell, J.R. (2012). Chemical perturbation of secondary metabolism demonstrates important links to primary metabolism. Chem. Biol. *19*, 1020–1027.

Czarny, T.L., and Brown, E.D. (2016). A small-molecule screening platform for the discovery of inhibitors of undecaprenyl diphosphate synthase. ACS Infect. Dis. *2*, 489–499.

Datsenko, K.A., and Wanner, B.L. (2000). One-step inactivation of chromosomal genes in *Escherichia coli* K-12 using PCR products. Proc. Natl. Acad. Sci. U S A *97*, 6640–6645.

Davis, N.K., and Chater, K.F. (1990). Spore colour in *Streptomyces coelicolor* A3(2) involves the developmentally regulated synthesis of a compound biosynthetically related to polyketide antibiotics. Mol. Microbiol. *4*, 1679–1691.

Eakin, A.E., Green, O., Hales, N., Walkup, G.K., Bist, S., Singh, A., Mullen, G., Bryant, J., Embrey, K., Gao, N., et al. (2012). Pyrrolamide DNA gyrase inhibitors: fragment-based nuclear magnetic resonance screening to identify antibacterial agents. Antimicrob. Agents Chemother. *56*, 1240–1246.

Fernandes, P., and Martens, E. (2017). Antibiotics in late clinical development. Biochem. Pharmacol. *133*, 152–163.

Flamm, R.K., Farrell, D.J., Rhomberg, P.R., Scangarella-Oman, N.E., and Sader, H.S. (2017). Gepotidacin (GSK2140944) activity against Gram-positive and Gram-negative bacteria. Antimicrob. Agents Chemother. *61*, e00468–17.

Fournier, B., Zhao, X., Lu, T., Drlica, K., and Hooper, D.C. (2000). Selective targeting of topoisomerase IV and DNA gyrase in *Staphylococcus aureus*: different patterns of quinolone-induced inhibition of DNA synthesis. Antimicrob. Agents Chemother. *44*, 2160–2165.

Gehrke, S.S., Kumar, G., Yokubynas, N.A., Côté, J.-P., Wang, W., French, S., MacNair, C.R., Wright, G.D., and Brown, E.D. (2017). Exploiting the sensitivity of nutrient transporter deletion strains in discovery of natural product antimetabolites. ACS Infect. Dis. *3*, 955–965.

Germe, T., Vörös, J., Jeannot, F., Taillier, T., Stavenger, R.A., Bacqué, E., Maxwell, A., and Bax, B.D. (2018). A new class of antibacterials, the imidazopyrazinones, reveal structural transitions involved in DNA gyrase poisoning and mechanisms of resistance. Nucleic Acids Res. *46*, 4114–4128.

Gibson, E.G., Bax, B., Pan, C.F., and Osheroff, N. (2019). Mechanistic and structural basis for the actions of the antibacterial gepotidacin against *Staphylococcus aureus* gyrase. ACS Infect. Dis. *5*, 570–581.

Haydon, D.J., Stokes, N.R., Ure, R., Galbraith, G., Bennett, J.M., Brown, D.R., Baker, P.J., Barynin, V.V., Rice, D.W., Sedelnikova, S.E., et al. (2008). An inhibitor of FtsZ with potent and selective anti-staphylococcal activity. Science *321*, 1673–1675.

Hooper, D.C., and Jacoby, G.A. (2016). Topoisomerase inhibitors: fluoroquinolone mechanisms of action and resistance. Cold Spring Harb. Perspect. Med. *6*, a025320.

Jani, C., Tocheva, E.I., McAuley, S., Craney, A., Jensen, G.J., and Nodwell, J. (2015). *Streptomyces*: a screening tool for bacterial cell division inhibitors. J. Biomol. Screen. *20*, 275–284.

Jia, B., Raphenya, A.R., Alcock, B., Waglechner, N., Guo, P., Tsang, K.K., Lago, B.A., Dave, B.M., Pereira, S., Sharma, A.N., et al. (2017). CARD

2017: expansion and model-centric curation of the comprehensive antibiotic resistance database. Nucleic Acids Res. *45*, D566–D573.

Kawai, Y., Moriya, S., and Ogasawara, N. (2003). Identification of a protein, YneA, responsible for cell division suppression during the SOS response in *Bacillus subtilis*. Mol. Microbiol. *47*, 1113–1122.

Khodursky, A.B., Zechiedrich, E.L., and Cozzarelli, N.R. (1995). Topoisomerase IV is a target of quinolones in *Escherichia coli*. Proc. Natl. Acad. Sci. U S A *92*, 11801–11805.

King, A.M., Reid-Yu, S.A., Wang, W., King, D.T., De Pascale, G., Strynadka, N.C., Walsh, T.R., Coombes, B.K., and Wright, G.D. (2014). Aspergillomarasmine A overcomes metallo-β-lactamase antibiotic resistance. Nature *510*, 503–506.

Kreiswirth, B.N., Löfdahl, S., Betley, M.J., O'Reilly, M., Schlievert, P.M., Bergdoll, M.S., and Novick, R.P. (1983). The toxic shock syndrome exotoxin structural gene is not detectably transmitted by a prophage. Nature *305*, 709–712.

Lahiri, S.D., Kutschke, A., McCormack, K., and Alm, R.A. (2015). Insights into the mechanism of inhibition of novel bacterial topoisomerase inhibitors from characterization of resistant mutants of *Staphylococcus aureus*. Antimicrob. Agents Chemother. *59*, 5278–5287.

McAuley, S., Huynh, A., Czarny, T.L., Brown, E.D., and Nodwell, J.R. (2018). Membrane activity profiling of small molecule growth inhibitors utilizing novel duel-dye fluorescence assay. MedChemComm *9*, 554–561.

McAuley, S., Vadia, S., Jani, C., Huynh, A., Yang, Z., Levin, P.A., and Nodwell, J.R. (2019). A chemical inhibitor of cell growth reduces cell size in *Bacillus subtilis*. ACS Chem. Biol. *14*, 688–695.

McCormick, J.R., and Flärdh, K. (2012). Signals and regulators that govern *Streptomyces* development. FEMS Microbiol. Rev. *36*, 206–231.

Monk, I.R., Shah, I.M., Xu, M., Tan, M.-W., and Foster, T.J. (2012). Transforming the untransformable: application of direct transformation to manipulate genetically *Staphylococcus aureus* and *Staphylococcus epidermidis*. MBio *3*, e00277–11.

Ogino, H., Teramoto, H., Inui, M., and Yukawa, H. (2008). DivS, a novel SOS-inducible cell-division suppressor in *Corynebacterium glutamicum*. Mol. Microbiol. *67*, 597–608.

Pimentel-Elardo, S.M., Sørensen, D., Ho, L., Ziko, M., Bueler, S.A., Lu, S., Tao, J., Moser, A., Lee, R., Agard, D., et al. (2015). Activity-independent discovery of secondary metabolites using chemical elicitation and cheminformatic inference. ACS Chem. Biol. *10*, 2616–2623.

Santos-Beneit, F., Roberts, D.M., Cantlay, S., McCormick, J.R., and Errington, J. (2017). A mechanism for FtsZ-independent proliferation in *Streptomyces*. Nat. Commun. *8*, 1378.

Santos-Beneit, F., Gu, J.Y., Stimming, U., and Errington, J. (2017). ylmD and ylmE genes are dispensable for growth, cross-wall formation and sporulation in *Streptomyces venezuelae*. Heliyon *3*, e00459.

Taylor, S.N., Morris, D.H., Avery, A.K., Workowski, K.A., Batteiger, B.E., Tiffany, C.A., Perry, C.R., Raychaudhuri, A., Scangarella-Oman, N.E., Hossain, M., and Dumont, E.F. (2018). Gepotidacin for the treatment of uncomplicated urogenital gonorrhea: a phase 2, randomized, dose-ranging, single-oral dose evaluation. Clin. Infect. Dis. *67*, 504–512.

Tehrani, K.H.M.E., and Martin, N.I. (2018). β-Lactam/β-lactamase inhibitor combinations: an update. MedChemComm *9*, 1439–1456.

Vos, S.M., Tretter, E.M., Schmidt, B.H., and Berger, J.M. (2011). All tangled up: how cells direct, manage and exploit topoisomerase function. Nat. Rev. Mol. Cell Biol. *12*, 827–841.

Zlitni, S., Blanchard, J.E., and Brown, E.D. (2009). High-throughput screening of model bacteria. Methods Mol. Biol. *486*, 13–27.

STAR★METHODS

KEY RESOURCES TABLE

REAGENT or RESOURCE	SOURCE	IDENTIFIER
Bacterial and Virus Strains		
Staphylococcus aureus	ATCC	ATCC 29213
Staphylococcus aureus	Kreiswirth et al., 1983	RN4220
Staphylococcus aureus	ATCC	ATCC BAA-1717
Staphylococcus aureus	ATCC	ATCC 33591
Staphylococcus aureus	Wright Clinical DB	C0117
Staphylococcus aureus	Wright Clinical DB	C0023
Staphylococcus aureus	Wright Clinical DB	C0112
Staphylococcus aureus	Wright Clinical DB	C0017
Staphylococcus aureus	Wright Clinical DB	C0032
Staphylococcus aureus	Wright Clinical DB	C0018
Staphylococcus aureus	Wright Clinical DB	C0019
Staphylococcus aureus	Wright Clinical DB	C0024
Escherichia coli MC1061	ATCC	ATCC 53338
Escherichia coli	Datsenko and Wanner, 2000	BW25113
Escherichia coli	King et al., 2014	ΔtolC ΔbamB
Streptomyces venezuelae	NRRL	NRRL B-65442
Chemicals, Peptides, and Recombinant Proteins		
Ciprofloxacin	Sigma-Aldrich	Cat#17850
Novobiocin	Sigma-Aldrich	Cat#N1628
Gepotidacin	MedChemExpress	Cat#HY-16742
Chloramphenicol	Sigma-Aldrich	Cat#C0378
Rifampicin	Sigma-Aldrich	Cat#R3501
Triclosan	Sigma-Aldrich	Cat#647950
Vancomycin	Sigma-Aldrich	Cat#V2002
Kanamycin	Sigma-Aldrich	Cat#B5264
CCCP	Sigma-Aldrich	Cat#C2759
Mitomycin C	Sigma-Aldrich	Cat#M4287
Bleomycin	Sigma-Aldrich	Cat#B1141000
Anhydrotetracycline	Sigma-Aldrich	Cat#A1200000
S.aureus Gyrase GyrASer84Leu	Inspiralis Inc.	Cat#SAAS84L01
Critical Commercial Assays		
GeneJET Genomic DNA Purification Kit	Thermo Scientific	Cat#K0721
E. coli Gyrase Supercoiling Kit	Inspiralis Inc.	Cat#K0001
S. aureus Gyrase Supercoiling Kit	Inspiralis Inc.	Cat#SAS4001
S.aureus Gyrase Cleavage Assay Kit	Inspiralis Inc.	Cat#SAGC001
E. coli Topo IV Decatenation Kit	Inspiralis Inc.	Cat#D4001
S.aureus Topo IV Decatenation Assay Kit	Inspiralis Inc.	Cat#SAD4001
Experimental Models: Cell Lines		
HEK293	CLS	CLS 300192
Oligonucleotides		
*gyrB*_K417E_F: ATATGGTACCACTCAGGATATGCCACAAATCT	This paper	N/A
gyrB_K417E_R: ATATGCGGCCGCATCGTGCAATAGACCATTTTGG	This paper	N/A

(*Continued on next page*)

Continued

REAGENT or RESOURCE	SOURCE	IDENTIFIER
Recombinant DNA		
Plasmid: pIMAY	Monk et al., 2012	Addgene 68939
Plasmid: pBR322	New England Biolabs	Cat#N3033S
Software and Algorithms		
Geneious	Biomatters	https://www.geneious.com/

CONTACT FOR REAGENT AND RESOURCE SHARING

Further information and requests for resources and reagents should be directed to and will be fulfilled by the Lead Contact, Justin Nodwell (justin.nodwell@utoronto.ca).

EXPERIMENTAL MODEL AND SUBJECT DETAILS

The experimental model that we have employed is *Staphyloccocus aureus* strain ATCC 29213. We have used the cell line HEK293, believed to be female in origin, as a control for EN-7 toxicity against human cells.

METHOD DETAILS

Strains, Media and Materials

S. venezuelae was grown on MYM (maltose, yeast extract, malt extract) agar, *B. subtilis* and *E. coli* strains were grown in LB media, *S. aureus* strains were grown in TSB (tryptic soy broth) and BHI (brain heart infusion) agar, and *E. faecalis* strains were grown in both BHI broth and agar.

Screen for Streptomyces Sporulation Inhibition

A lawn of *S. venezuelae* spores was plated on MYM agar plates and allowed to dry. For the initial screen, 2 μL of each molecule at 2.5 mM was spotted directly to the agar plate. For known antibiotics or follow-up screening, 10 μL of molecule was added to a drug disk, allowed to dry, and placed on the agar plate. The *S. venezuelae* plate was incubated at 30°C for 48 hours and photographed. The *whi* phenotype was identified by eye. Hits were selected based on their ability to induce a *whi* phenotype at sub-inhibitory concentrations and scored by eye on the size of the induced *whi* region and the zone of inhibition.

Scanning Electron Microscopy

To prepare samples for SEM, the agar plate containing a lawn of *S. venezuelae* was cut at the edge of the zone of inhibition and fixed with 2% glutaraldehyde in 0.1% sodium cacodylate buffer followed by sputter coating with gold. Images were obtained and processed using a FEI XL30 ESEM. The contrast of the resulting images was adjusted to highlight the appearance or lack of spore formation.

Minimum Inhibitory Concentration

The minimum inhibitory concentration (MIC) defines the lowest concentration of a compound required to inhibit the growth. A single colony of the bacteria being tested was grown in 5 mL of liquid growth media. Following overnight growth, the culture was diluted 100-fold into fresh media and incubated at 37°C until the cells reached mid-log phase (OD_{600} of 0.3 to 0.5). The cells were diluted 10,000-fold into fresh media and the MIC determined for each of the strains by adding 2 μL of the diluted compound to 198 μL of the diluted cells in a clear 96-well plate. The plates were incubated at 37°C overnight and the optical density at 600 nm was measured for each well. All MIC assays were performed in triplicate.

Isolation of EN-7 Resistant Mutants

Resistant mutants were generated using both serial passaging and spontaneous mutant methods. For serial passaging, a single colony of *S. aureus* ATCC 29213 was incubated overnight in TSB liquid media at 37°C. Following overnight growth, 2 μL of the overnight culture was diluted into 5 mL of fresh media containing 0.25X MIC of EN-7 and incubated overnight at 37°C. The culture was then diluted into 0.5X MIC EN-7 and the procedure repeated with each subsequent dilution increasing the EN-7 concentration by 2X until reaching 16X MIC of EN-7. This culture was then plated on solid TSB agar and a single colony selected as the mutant strain. For spontaneous mutants, approximately 10^8 *S. aureus* cells from an overnight culture were plated to BHI agar plate containing 10 μM (10X MIC) EN-7 and incubated overnight at 37°C. The resistance mutation frequency was determined by counting the number of colonies on the EN-7 plates versus the number of colonies of the untreated plates.

Sequencing of Resistant Mutants

Genomic DNA was isolated from overnight cultures of the mutant *S. aureus* strains using a GeneJET Genomic DNA Purification Kit from Thermo Scientific. Briefly, 2 mL of overnight culture of *S. aureus* was harvested by centrifuging at 5000 g for 10 minutes. The pellet was resuspended in lysis buffer (20 mM Tris-HCl, pH 8.0, 2 mM EDTA, 1.2% Triton X-100) with 2 μg/mL lysostaphin and incubated in a water bath at 37°C for 30 minutes. Following incubation, 200 μL of lysis solution with 20 μL Proteinase K was added to each sample and incubated at 56°C for another 30 minutes. Following lysis, 20 μL RNaseA solution was added and allowed to incubate at room temperature for 10 minutes. Addition of 400 μL of 50% ethanol was then followed by DNA isolation and purification on a GeneJET Genomic DNA Purification Column.

Sequencing was performed using an Illumina MiSeq with Nextera XT library preparation and analyzed with Geneious software to map the reads to the *S. aureus* ATCC 29213 assembly (RefSeq GCF_001879295.1) and identify SNPs.

Confirming Resistance Mutations through Allelic Exchange in *S. aureus*

The *gyrB* sequence containing the K417E mutation was amplified from EN-7 resistant *S. aureus* mut-1 genomic DNA from the using primers *gyrB*_K417E_F (ATATGGTACCACTCAGGATATGCCACAAATCT) and gyrB_K417E_R (ATATGCGGCCGCATCGTGCAATA GACCATTTTGG). The amplicon was digested using Fast Digest KpnI and NotI and ligated into the similarly cut pIMAY plasmid. The pIMAY gyrB_K417E plasmid was passaged through *S. aureus* RN4220 at 30°C before being electroporated into ATCC 29213 and also grown at 30°C. A single colony containing the pIMAY plasmid as well as a colony of wild-type *S. aureus* ATCC 29213 were selected and grown separately overnight in 5 mL TSB broth. 100 μL of the overnight cultures were plated to separate 10 mL BHI agar plates containing 1 μg/mL anhydrotetracycline and 10 μM EN-7 and incubated overnight at 37°C. This was to ensure a single-step allelic exchange in the plasmid containing culture. Anhydrotetracycline is used to activate the anti-*secY* sequence in the plasmid and the temperature-sensitive origin of replication prevents replication at 37°C. To confirm that the pIMAY plasmid had been lost, 24 colonies from the transformation plates were replicate plated to a BHI plate containing 10 μM EN-7 and another plate containing both 10 μM EN-7 and 25 μg/mL chloramphenicol, the antibiotic used to select for pIMAY containing cells. The confirm the allelic exchange introduced the single mutation, colony PCR was performed on eight of the 24 colonies using both *gyrB*_K417E_F and *gyrB*_K417E_R primers as well as *gyrA*_A34T_F (ATATGGTACCTGCACAGCCACCGTTGTATA) and *gyrA*_P219Q_R (ATATGCGGCCGCTGTGGGCACGATCTTTAGCT) primers. These amplicons were sequenced using an ABI 3730XL instrument.

E. coli Gyrase Supercoiling

1 U of DNA gyrase was incubated with 0.5 μg of relaxed pBR322 DNA in a 30 μl reaction at 37°C for 30 minutes under the following conditions: 35 mM Tris.HCl (pH 7.5), 24 mM KCl, 4 mM $MgCl_2$, 2 mM DTT, 1.8 mM Spermidine, 1 mM ATP, 6.5% (w/v) glycerol and 0.1 mg/mL BSA. Each reaction was stopped by the addition of 30 μl chloroform/iso-amyl alcohol (24:1) and 20 μl Stop Dye (40% sucrose, 100 mM Tris.HCl (pH 7.5), 10 mM EDTA, 0.5 μg/mL bromophenol blue), before being loaded on a 1.0% TAE (Tris.acetate 0.04 mM, EDTA 0.002 mM) agarose gel. Gels run at 90 V for 2 hours. Bands were visualized by ethidium staining for 20 minutes and destaining in water for 20 minutes. Gels were scanned using a gel documentation system (GeneGenius, Syngene, Cambridge, UK) and percent inhibition levels obtained with gel scanning software (GeneTools, Syngene, Cambridge,UK).

S. aureus Gyrase Supercoiling

As for *E. coli* gyrase except assay conditions were: 40 mM HEPES. KOH (pH 7.6), 10 mM magnesium acetate, 10 mM DTT, 2 mM ATP, 500 mM potassium glutamate, and 0.05 mg/mL BSA. Gels run at 80 V for 3 hours.

E. coli Topoisomerase IV Decatenation

1 U of topoisomerase IV was incubated with 200 ng kDNA DNA in a 30 μl reaction at 37 C for 30 minutes under the following conditions: 50 mM HEPES-KOH (pH 7.6), 100 mM potassium glutamate, 10 mM magnesium acetate, 10 mM dithiothreitol, 1 mM ATP and 50 μg/mL BSA. Each reaction was stopped by the addition of 30 μl chloroform/iso-amyl alcohol (24:1) and 20 μl Stop Dye, before being loaded on a 1.0% TAE agarose gel. Gels run at 90 V for 2 hours.

S. aureus Topoisomerase IV Decatenation

As for E coli topoisomerase IV except assay conditions were: 50 mM Tris-HCl (7.5), 5 mM $MgCl_2$, 5 mM DTT, 1.5 mM ATP, 350 mM potassium glutamate and 0.05 mg/mL BSA. Gels run at 80 V for 2 hours.

S. aureus Gyrase Cleavage

1 U of gyrase was incubated with 0.5 μg of supercoiled pBR322 DNA in a 30 μL reaction at 37 C for 30 minutes under the following conditions: 40 mM HEPES. KOH (pH 7.6), 10 mM magnesium acetate, 10 mM DTT, 100 mM potassium glutamate and 0.05 mg/mL albumin. 0.2 % SDS and 0.1 mg/mL Proteinase K were added before a further incubation at 37°C for 30 minutes. Each reaction was stopped by the addition of 30 μL chloroform/iso-amyl alcohol (24:1) and 20 μL Stop Dye, before being loaded on a 1.0% TAE agarose gel. Gels run at 80 V for 2 hours.

S. aureus Gyrase Relaxation

1 U of gyrase was incubated with 0.5 μg of supercoiled pBR322 DNA in a 30 μL reaction at 37°C for 60 minutes under the following conditions: 50 mM Tris.HCl (pH 7.5), 5 mM MgCl2,5 mM DTT, 350 mM potassium glutamate, and 0.05 mg/mL albumin. Each reaction was stopped by the addition of 30 μL chloroform/iso-amyl alcohol (24:1) and 20 μl Stop Dye, before being loaded on a 1.0% TAE agarose gel. Gels run at 80 V for 2 hours.

Synthesis and Characterization of EN-7

Compound EN-7 was synthesized by Wuxi PharmaTech, China, from commercially available 5-phenyl isoxazole-3-carboxylic acid (5) and quinolyl 8-carboxaldehyde (1), via the intermediates compounds 2, 3, and 4, using the route shown in Figure S5. A mixture of Compound **1** (4 g, 25.45 mmol, 1 eq) and ethyl 2-(triphenyl-phosphanylidene)acetate (8.87 g, 25.45 mmol, 1 eq) in toluene (40 mL) was stirred at 100°C for 6 h. LCMS showed compound **1** was consumed and the desired mass was detected. The mixture was concentrated to obtain the residue which was purified by column chromatography (SiO2, petroleum ether: ethylacetate =10:1 to 3:1). Compound **2** (4 g, crude) was obtained as a yellow solid. To compound **2** (1.00 g, 4.40 mmol, 1.00 eq) in a solution of EtOH (6 mL) and H2O (2 mL) was added LiOH•H2O (923.26 mg, 22.00 mmol, 5.00 eq) and the mixture was stirred at 25°C for 2 h. LCMS showed the reactant 1 was consumed and the desired mass was detected. The solution was poured into water (20 mL), and the aqueous phase was extracted with ethyl acetate (10 mL × 3). The pH of aqueous phase was adjusted to 3 by HCl (4 M), then the precipitate was collected and concentrated *in vacuo*. The mixture was used in the next step without purification. Compound **3** (500 mg, crude) was obtained as a white solid. To a solution of compound **5** (500.00 mg, 2.64 mmol, 1.00 eq) and DMF (1.93 mg, 26.43 μmol, 2.03 μL, 0.01 eq) in THF (10 mL) was added $(COCl)_2$ (503.23 mg, 3.96 mmol, 347.05 μL, 1.50 eq), drop-wise, at 0°C, then the mixture was stirred at 25°C for 1 h. $NH_2NH_2 \cdot H_2O$ (2.5 mL) was added, drop-wise, into the mixture at 0°C, then stirred at 25°C for 1 h. LCMS showed compound **5** was consumed and the desired mass was detected. The residue was poured into water (100 mL) and the aqueous phase was extracted with ethyl acetate (20 mL × 3). The combined organic phase was washed with brine (10 mL), dried with anhydrous Na_2SO_4, filtered and concentrated *in vacuo*. The residue was used to the next step without purification. Compound **4** (200 mg, crude) was obtained as a pink solid. To a mixture of compound **3** (80.00 mg, 401.60 μmol, 1.00 eq), Compound **4** (81.60 mg, 401.60 μmol, 1.00 eq), EDCI (76.99 mg, 401.60 μmol, 1.00 eq) in Pyridine (1 mL) was stirred at 50°C for 12 h. LCMS showed Compound **4** was consumed and the desired mass was detected. The mixture was concentrated to obtain the residue, to which was added water (10 mL) and ethyl acetate (5 mL) and the precipitate was collected to obtain the crude product. To the crude product was added MeOH (4 mL) and DMSO (2 drops) and the mixture was stirred at 40°C for 2 h. After filtration, the filter cake was washed with MeOH (5 mL) and concentrated to get the product. Compound EN-7 (40 mg, 97.82 μmol, 24.36% yield, 94% purity) was obtained as a white solid. **MS** (ESI): 385.1 (M+H)+ ^{1}**H NMR** (400 MHz, DMSO-d6) δ= 10.89 (*br s*, 1H), 10.43 (*br s*, 1H), 9.03 (*br d*, J=2.7 Hz, 1H), 8.78 (*d*, J=16.1 Hz, 1H), 8.46(*br d*, J=8.1 Hz, 1H), 8.18 – 8.05 (*m*, 2H), 7.98 (*br d*, J=5.7 Hz, 2H), 7.72 (*br t*, J=7.6 Hz, 1H), 7.65 (*dd*, J=4.1, 8.3 Hz, 1H), 7.61- 7.55 (*m*, 3H), 7.49 (*s*, 1H), 7.08 (*d*, J=16.1 Hz, 1H)

QUANTIFICATION AND STATISTICAL ANALYSIS

Error was calculated for all measurements in order to confirm that differences that were measured in the presence and absence of antibiotic are statistically meaningful. For example, in Figures 3, 5, and S3, error bars on the absorbance values are derived from at least three replicates. All reported measurements were found to be statistically significant by the criterion of non-overlapping error. We used Prism to make the x-y plots, Pymol for the protein structures, and Adobe Illustrator for the figure layouts.

DATA AND CODE AVAILABILITY

All sequence alterations discovered in the *Staphylococcus* genome mutants are reported in this paper: there are no other significant deviations from the reported genomes. The kits that we have used for *in vitro* gyrase assays (supercoiling and decatenation) are commercially available from Inspiralis Inc. (e.g. catalogue #s Cat#SAAS84L01, Cat#K0001, Cat#SAS4001, Cat#SAGC001, Cat#D4001, Cat#SAD4001). Any of the raw data used in this work will be made available upon request.

Cell Chemical Biology, Volume *26*

Supplemental Information

Discovery of a Novel DNA Gyrase-Targeting Antibiotic through the Chemical Perturbation of *Streptomyces venezuelae* Sporulation

Scott McAuley, Alan Huynh, Alison Howells, Chris Walpole, Anthony Maxwell, and Justin R. Nodwell

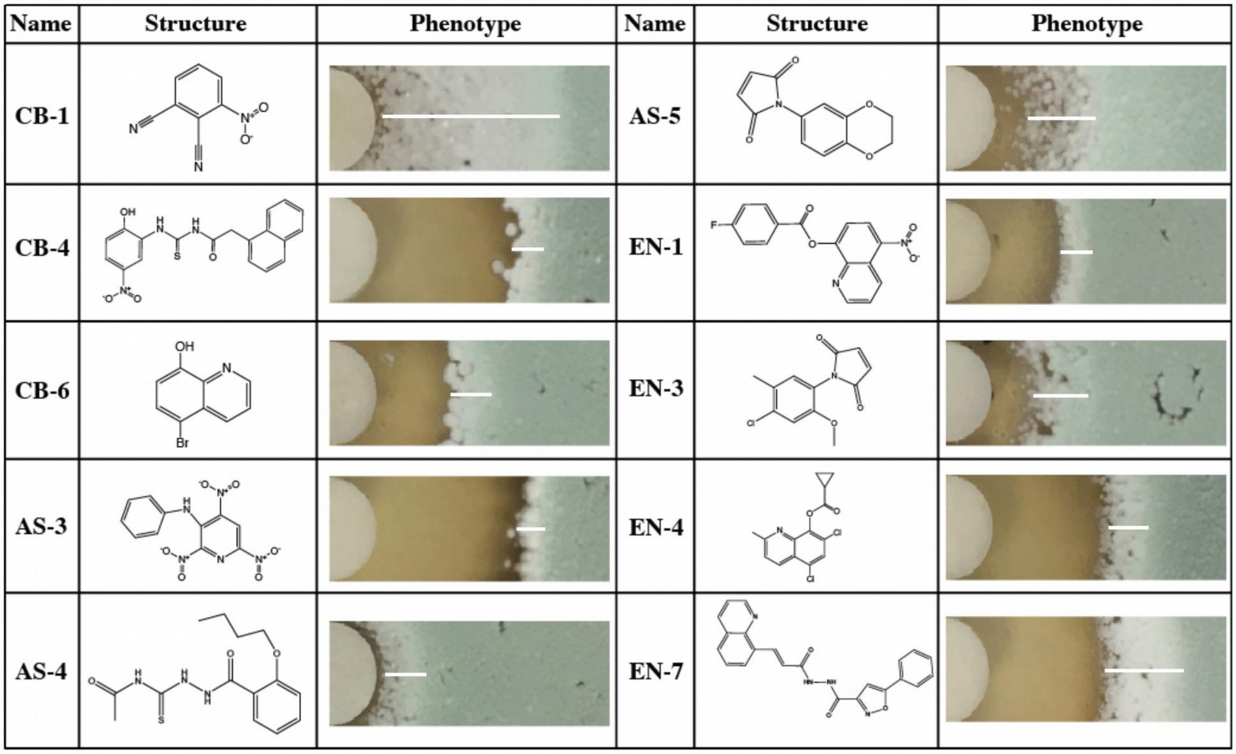

Figure S1, related to Figure 1. Chemical structures and inhibition of *S. venezuelae* sporulation by the screening hits. 10 µL of each molecule was added to the drug disk, placed on a lawn of *S. venezuelae* spores and incubated at 30 ^{o}C for 48 hours before imaging. The EN-1 and AS-5 stocks were 10 mM while the remaining were at 2.5 mM. A white horizontal line has been added for visual reference.

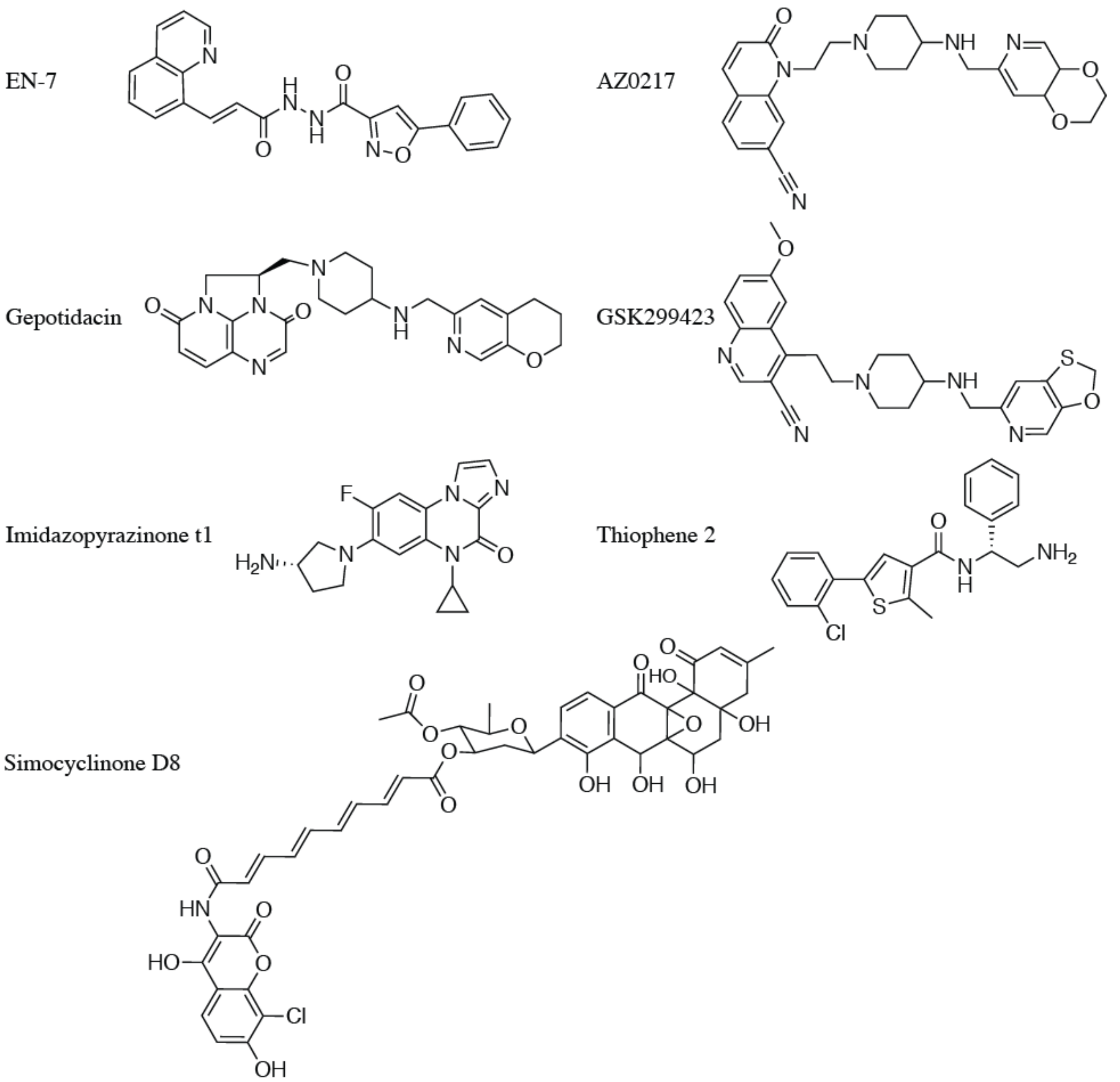

Figure S2, related to Figure 2. Chemical structures of EN-7 with other investigational gyrase inhibitors

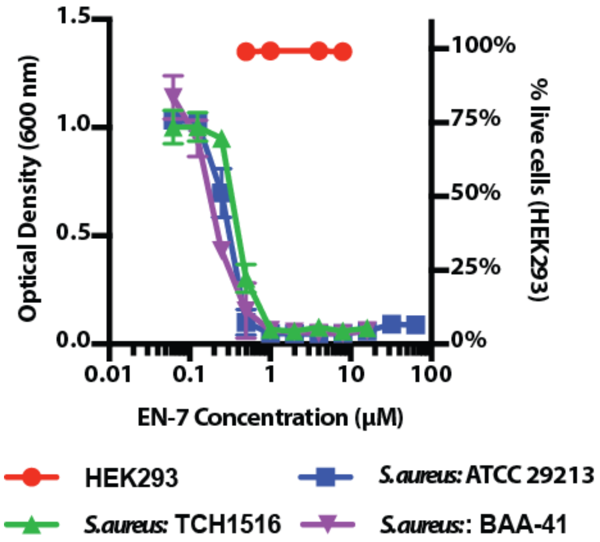

Figure S3, related to Table 1. EN-7 inhibits growth of Gram-positive *S. aureus* while having no effect on human cell lines, such as HEK293.

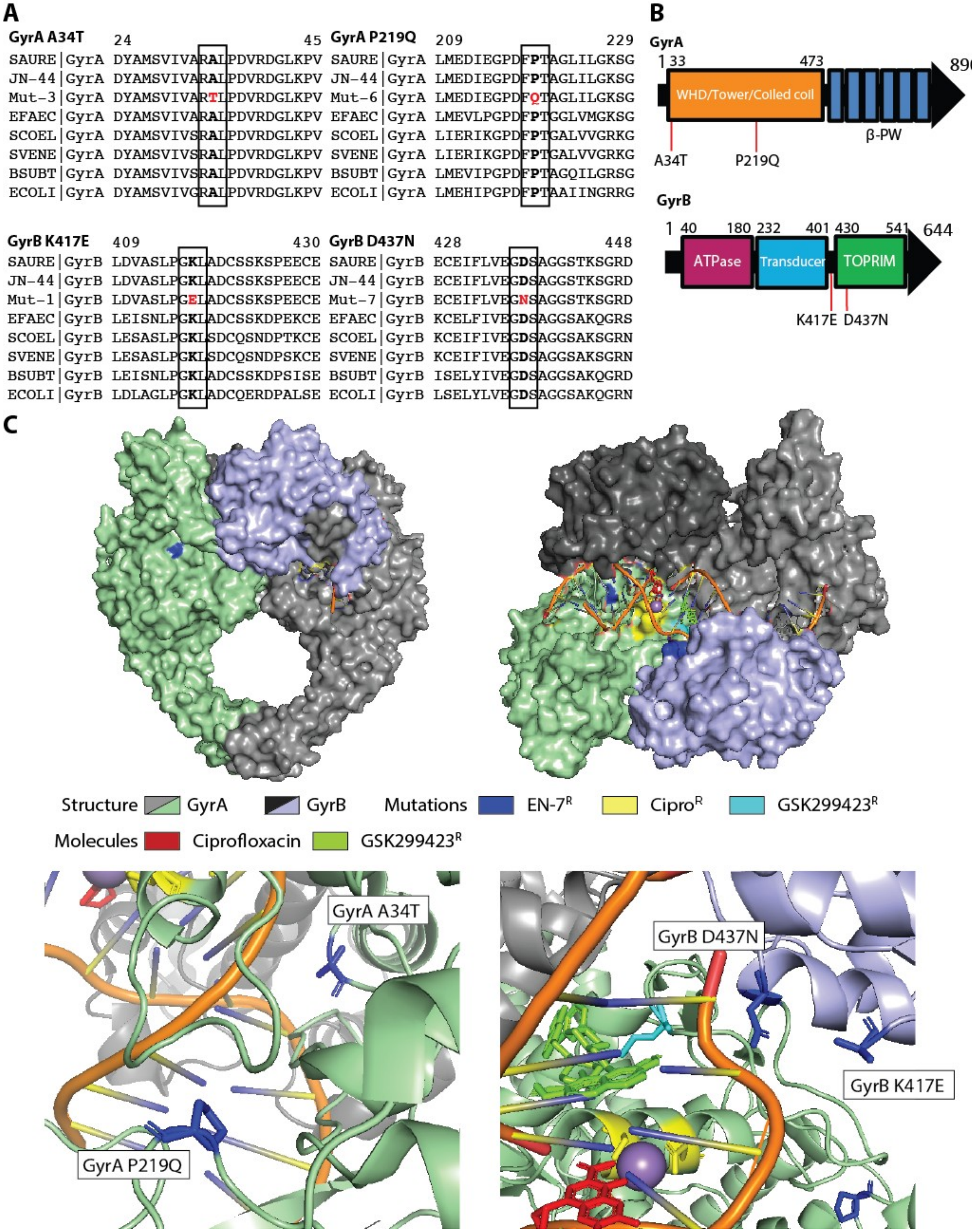

Figure S4, related to Table 2. Analysis of EN-7-resistance gyrase mutations. (A) Amino acid alignment of the two GyrA and two GyrB mutations found in the EN-7 resistant *S. aureus* strains against published *S. aureus* (SAURE), parent strain (JN-44), *Enterococcus faecalis* (EFAEC), *Streptomyces coelicolor* (SCOEL),

Streptomyces venezuale (SVENE), *Bacillus subtilis* (BSUBT), and *Escherichia coli* (ECOLI) sequences. (B) Location of EN-7 resistant mutations on linear GyrA and GyrB domain structure. (C) Location of EN-7 resistant mutations mapped to a published gyrase crystal structure. The structural information was created by overlaying a *S. aureus* gyrase structure with GSK299423 (2XCS) and a *S. aureus* gyrase structure with ciprofloxacin (2XCT). Ciprofloxacin is depicted in the structure as red while GSK299423 is green. Resistance determinants for EN-7 (blue), ciprofloxacin (yellow), and GSK299423 (teal) are also depicted.

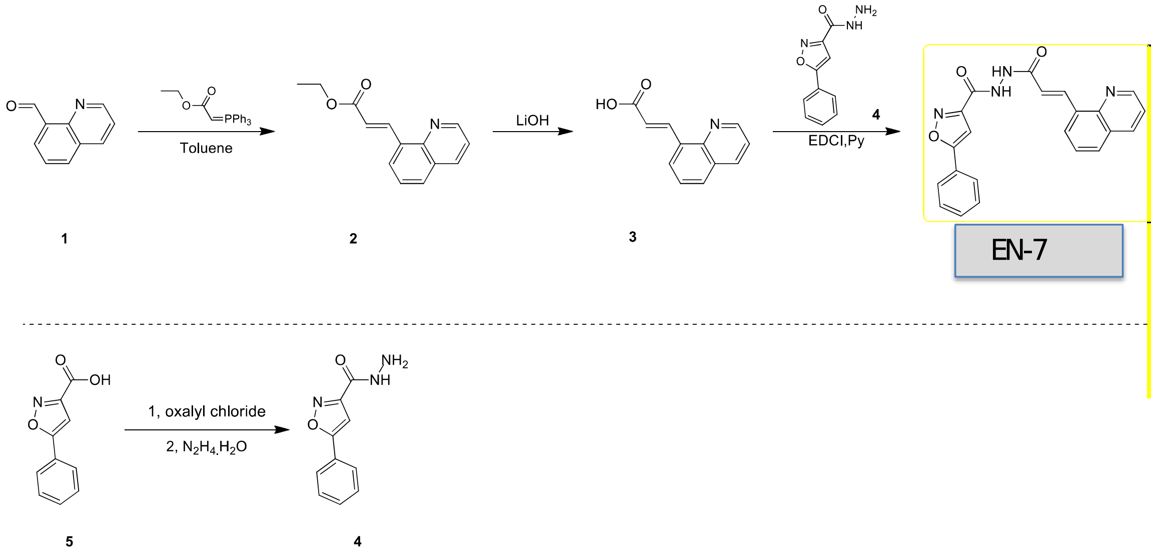

Figure S5, related to STAR Method, Synthesis and characterization of EN-7

Strain	C0117	C0023	C0112	C0017	C0032	C0018	C0019	C0024		
**MIC (μM)**	> 128	1	4	1	> 128	> 128	1	1		
**Resistance elements**									**Product**	**Inhibited antibiotics**
parE P585S	■						■		Modified topoisomerase IV	No reference
ErmA	■	■	■	■					Ribosomal RNA methyltransferase	Macrolides
ANT(4')-Ib	■	■	■	■					Nucleotidyltransferase	Aminoglyclosides
ANT(9)-Ia	■	■	■	■					Nucleotidyltransferase	Aminoglyclosides
blaZ			■	■	■	■	■		Beta-lactamases	Beta-lactams
SAT-4					■	■			Acetyltransferase	Nucleosides
mphC					■	■			Phosphotransferase	Macrolides
APH(3')-IIIa					■	■			Phosphotransferase	Macrolides
bcrA					■	■			ABC efflux pump	Bacitracin
gyrB K66Q					■	■	■	■	Modified gyrase	No reference
gyrA R246C			■						Modified gyrase	No reference
gyrA T457A		■				■	■	■	Modified gyrase	No reference
gyrA D459N		■				■	■	■	Modified gyrase	No reference
gyrA S85P		■		■					Modified gyrase	Fluoroquinolones
gyrA S84L	■	■	■	■	■	■	■	■	Modified gyrase	Fluoroquinolones
mecI	■	■	■	■	■	■	■		PBP2A	Beta-lactams
mprF	■	■	■	■	■	■	■	■	Modified phosphatidylglycerol	Daptomycin
tet38	■	■	■	■	■	■	■	■	MFS efflux pump	Tetracycline
Sav1866	■	■	■	■	■	■	■	■	ABC efflux pump	Multidrug
norB	■	■	■	■	■	■	■	■	MFS efflux pump	Fluoroquinolones & Tetracycline
norA	■	■	■	■	■	■	■	■	MFS efflux pump	Fluoroquinolones & Tetracycline
murA	■	■	■	■	■	■	■	■	UDP-N-acetylglucosamine enolpyruvyl transferase	Fosfomycin
mgrA	■	■	■	■	■	■	■	■	MFS efflux pump	Multidrug
mepR	■	■	■	■	■	■	■	■	MATE transporter	Tetracycline
mepA	■	■	■	■	■	■	■	■	MATE transporter	Tetracycline
mecR1	■	■	■	■	■	■	■	■	PBP2A	Beta-lactams
mecC	■	■	■	■	■	■	■	■	PBP2A	Beta-lactams
lmrB	■	■	■	■	■	■	■	■	ABC efflux pump	Lincosamides
EF-Tu	■	■	■	■	■	■	■	■	Elongation factor Tu	Kirromycin
cls	■	■	■	■	■	■	■	■	Cardiolipin synthetase	Daptomycin
dfrC	■	■	■	■	■	■	■	■	Dihydrofolate reductase	Trimethoprim
ileS	■	■	■	■	■	■	■	■	Isoleucyl-tRNA synthetase	Mupirocin
arlS	■	■	■	■	■	■	■	■	MFS efflux pump	Fluoroquinolones
alaS	■	■	■	■	■	■	■	■	Alanyl-tRNA synthetase	Novobiocin
arlR	■	■	■	■	■	■	■	■	MFS efflux pump	Fluoroquinolones

Table S1, related to Table 1. EN-7 inhibits growth of extensively resistant *S. aureus* clinical strains. Blue boxes indicates that the corresponding antibiotic resistance gene was found within the clinical strain's genome. A white box indicates that the gene was absent in the genome.
